# Scalable screening of ternary-code DNA methylation dynamics associated with human traits

**DOI:** 10.1016/j.xgen.2025.100929

**Published:** 2025-07-03

**Authors:** David C. Goldberg, Cameron Cloud, Sol Moe Lee, Bret Barnes, Steven Gruber, Elliot Kim, Anita Pottekat, Maximillian S. Westphal, Luana McAuliffe, Elisa Majounie, Manesh Kalayil Manian, Qingdi Zhu, Christine Tran, Mark Hansen, Jelena Stojakovic, Jared B. Parker, Rahul M. Kohli, Rishi Porecha, Nicole Renke, Wanding Zhou

**Affiliations:** 1 Center for Computational and Genomic Medicine, The Children’s Hospital of Philadelphia, Philadelphia, PA 19104, USA; 2 Illumina, Inc., San Diego, CA 92122, USA; 3 Department of Medicine, University of Pennsylvania, Philadelphia, PA 19104, USA; 4 Department of Pathology and Laboratory Medicine, University of Pennsylvania, Philadelphia, PA 19104, USA

**Keywords:** DNA methylation, epigenetics, epigenome-wide association study, 5-hydroxymethylation, epigenetic clock, Infinium array

## Abstract

Epigenome-wide association studies (EWASs) are transforming our understanding of the interplay between epigenetics and complex human traits. We introduce the methylation screening array (MSA) to enable scalable and quantitative screening of trait-associated DNA cytosine modifications in large human populations. The MSA integrates EWASs and cell-type-linked methylation signatures, covering diverse traits and diseases. Using the MSA to profile the ternary-code DNA methylations—dissecting 5-methylcytosine (5mC), 5-hydroxymethylcytosine (5hmC), and unmodified cytosine—revealed a previously unappreciated role of 5hmC in mediating human trait associations and epigenetic clocks. We demonstrated that 5hmCs complement 5mCs in defining epigenetic cell identities. In-depth analyses highlighted the cell-type context of EWAS and genome-wide association study (GWAS) hits. Targeting aging, we uncovered shared and tissue-specific 5hmC aging dynamics and tissue-specific rates of mitotic hyper- and hypomethylation. These findings chart a landscape of the complex interplay of the two forms of cytosine modifications in diverse human tissues and their roles in health and disease.

## Highlights

• The methylation screening array enables scalable profiling of human epigenomes
• Base resolution atlas of matched 5modC and 5hmC profiles across human tissues
• Determination of 5modC/5hmC and gene expression patterns regulating tissue identity
• EWAS identifies roles of 5hmCs in aging and epigenetic clocks

## Graphical abstract

**Figure:**
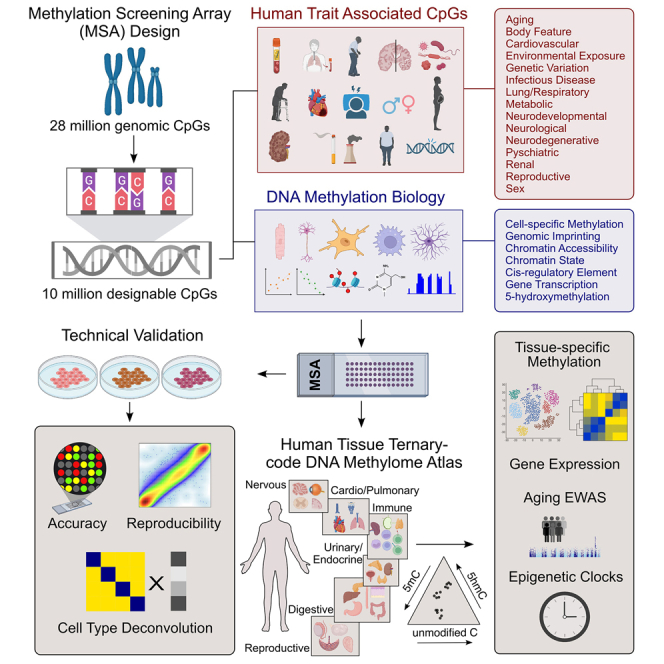

## Teaser

Goldberg et al. develop the methylation screening array for profiling large human populations for trait associations. They generate and analyze a 5mC-5hmC methylation atlas across human tissue, revealing the epigenetic context of EWAS and GWAS loci and highlighting overlooked roles of 5hmCs in tissue identity, transcriptional regulation, and epigenetic aging.

## Introduction

The dynamic genome-wide patterns of cytosine modifications, including 5-methylcytosine (5mC), 5-hydroxymethylcytosine (5hmC), and unmodified cytosine (C) (collectively referred to as the ternary-code methylation pattern), play a critical role in regulating gene expression,^1^ genome stability maintenance,^2^ and organismal development.^3^ Through these roles, DNA methylation has been extensively associated with cellular and physiological human traits^4^ and is increasingly utilized as a biomarker in translational research and clinical applications.^5^^,^^6^ Notable examples include applying DNA methylation to classify cancer and rare diseases,^7^^,^^8^^,^^9^^,^^10^ liquid biopsy-based disease diagnosis,^11^ and assessing disease hazards through methylation risk scores^12^ and forensic analysis.^13^ Analysis of DNA methylation profiles is also crucial for elucidating gene transcription mechanisms,^14^ understanding cell identity maintenance,^15^ studying variations in cell composition,^16^ and investigating gene-environment interactions within populations.^4^

Epigenome-wide association studies (EWASs) investigate large human populations to uncover how DNA cytosine modifications are associated with human traits and diseases.^4^^,^^17^^,^^18^ Over the past decade, EWAS has been instrumental in uncovering links between DNA methylation and diverse human phenotypes. To support these studies, methodologies developed to profile DNA methylation across the genome^19^ are often challenged by the large size of the human genome, the complex DNA methylation biology across genomic regions, and prevalent inter-cellular heterogeneity in tissues.^20^ The most comprehensive DNA methylation profiling assay is single-cell whole-genome methylation sequencing (scWGMS), which offers unparalleled detail by providing base-resolution data for individual cells.^21^ However, the high costs and technical complexity of scWGMS often restrict its use to a limited number of samples.^22^ As it is currently not practical to implement scWGMS for population studies, alternative methodologies are more frequently used, trading off genome coverage, base resolution, or cell-type resolution to reduce costs and technical demands. These include methods for profiling bulk tissues^23^ or fluorescence-activated cell sorting (FACS)-purified cells (e.g., bulk deep WGBS or nanopore sequencing),^24^ targeted genome capture (e.g., reduced representation bisulfite sequencing [RRBS]^25^), and the use of data techniques to interpret sparse signals (e.g., low-pass sequencing^26^).

The Infinium DNA methylation BeadChip has been a robust solution for large-scale methylation discovery and screening efforts due to its ease of experiment and data analysis,^27^ base-resolution detection, and high quantitative granularity. This platform has been central to consortia such as The Cancer Genome Atlas (TCGA) and has amassed over 80,000 HM450 methylomes^28^ and a comparable number of EPIC array methylation profiles in the Gene Expression Omnibus (GEO). While sequencing-based methods are more commonly used for case-specific and mechanistic studies, Infinium arrays are often preferred in discovering population-scale trait associations, including methylation quantitative trait loci (meQTL) studies,^29^^,^^30^ epigenetic risk scoring,^31^^,^^32^ and EWASs in humans^33^^,^^34^ and other mammalian species.^35^^,^^36^^,^^37^ Such adoption is partly due to the need for population studies to cover a large number of samples to dissect multiple cohort covariates (e.g., sex, age, genetic background, and tissue type) and their interactions, and, in others, it is due to the high depths required to capture nuanced variations in cytosine modification levels.^38^^,^^39^ A prominent example is 5hmCs, which are inherently stochastic—often under 30% per site, even in homogeneous cell populations,^40^ unlike the bimodal distribution typical of 5mCs—and are concentrated in specific regulatory regions,^41^^,^^42^ necessitating high quantitative resolution for accurate measurements on a small number of sites rather than sparse whole-genome coverage.

**Figure 1:**
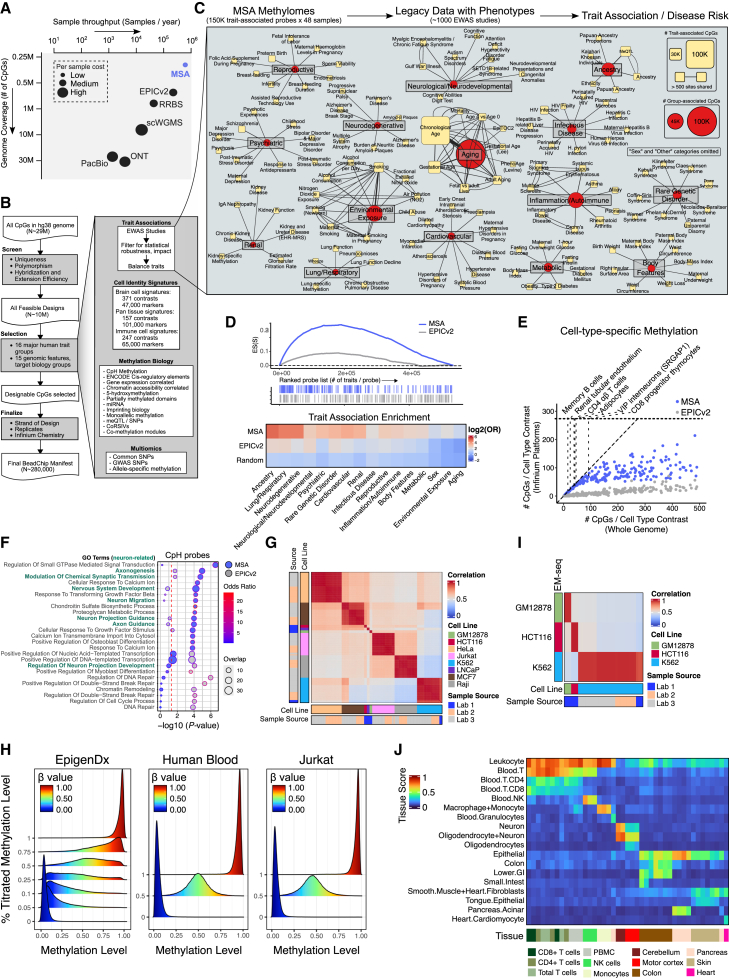
MSA design, trait representation, and benchmarking (A) Overview of methylation technologies across genome coverage, cost, and throughput. (B) MSA design schematic illustrating the design process. From the designable probe pool (left), CpGs whose methylations are associated with diverse methylation biology and human traits were identified (right). (C) Major trait categories (red) and representative sub-traits (yellow) included in MSA; some traits may appear multiple times due to cohort differences. (D) Top: MSA and EPICv2 probe enrichment EWAS hits ranked by the number of trait associations. Bottom: heatmap showing the enrichment (log_2_ odds ratio) of major trait group probes on MSA vs. EPICv2 and random Infinium probes. (E) Number of CpGs per cell-type contrast on MSA vs. EPICv2 for contrasts with <500 high-quality whole-genome markers. (F) Gene Ontology (GO) term enrichment (hypergeometric test) for genes linked to CpH probes (minimum two probes per gene) on MSA and EPICv2. (G) Heatmap of beta value correlations between cell lines profiled by MSA. “Sample source” indicates the culturing lab. (H) Density plots of measured beta values for methylation titration standards. (I) Heatmap of beta value correlations between MSA (columns) with EM-seq (row) profiles for the same cell line samples. “Sample source” indicates culturing lab. (J) Tissue prediction scores using an EPIC prediction model on MSA tissue profiles (columns). Missing EPIC probes were substituted with MSA nearest-neighbor probes.

Array technologies rely on static probe designs that fix the CpG space to those selected during the array’s development.^43^ While this permits cross-study comparisons, the current design has the following limitations. First, WGMS of 5mCs and 5hmCs in human cells and tissues has significantly advanced our understanding of cell-type methylation at high resolutions^24^ since the last human array design.^44^ Current EPICv2 arrays, largely inheriting EPIC, have yet to incorporate the recent discoveries (e.g., of 5hmCs).^22^^,^^24^^,^^38^^,^^39^ Further, most predictive models based on existing arrays hinge on a small number of trait associates. For example, most epigenetic clock models used hundreds of CpGs and reached high prediction accuracy.^45^ Minimalistic approaches were taken in epigenetic clock construction,^46^ cell-type deconvolution,^47^ and cancer classification.^48^ These observations motivate the notion that building compatible but condensed arrays for applying existing models and reassessing associations in significantly larger, more inclusive, and stratified human populations should be feasible (Figure 1A).

To implement these thoughts, we present the rationale, systematic design, and the first application of the methylation screening array (MSA), the latest Infinium BeadChip iteration. Compared to previous Infinium BeadChips, the MSA has concentrated its coverage on trait-associated methylation (∼5.6 trait associations per site vs. ∼2.2 in EPICv2; STAR Methods) and cell-identity-associated methylation variations (∼3.7 cell signatures per site vs. ∼2.3 in EPICv2, with an additional 48 cell-type contrasts). Half of the design targeted previously reported EWAS associations. The other half leverages the latest single-cell and bulk whole-genome methylation profiling efforts that deeply characterize diverse human cell types. This dual approach enables high-resolution cell-type deconvolution, supported by reference methylation panels and predictive models that we have rigorously benchmarked in this study. Compared to the 8-sample plate design used in previous methylation arrays, the MSA is built on a novel 48-sample EX methylation platform to achieve ultra-high sample throughput at a lower cost per sample while screening for more traits per probe. Evaluation of the array’s accuracy and reproducibility confirms its robustness for population-scale applications. We also demonstrate the capacity for 5hmC profiling using a bisulfite APOBEC-coupled epigenetic sequencing (bACE) protocol that combines bisulfite conversion with further ABOBEC3A deamination. Applying the MSA to various human tissues, we characterize tissue-specific 5mC and 5hmC genomic distribution and demonstrate the capacity for accurate cell-type deconvolution. We performed an EWAS for 5hmC in aging and sex and identified previously under-reported contributions of 5hmC to the prediction mechanism of epigenetic clocks. Analysis of 64 whole-blood methylomes demonstrated variable methylation at established EWAS loci and age- and sex-related immune cell composition alterations across the lifespan.

## Results

### Systematic design of MSA

We designed the MSA by consolidating human trait-associated loci from previous EWASs with novel probe designs targeting diverse methylation biology (Figure 1B; Table S1). Following quality control, the MSA contains 284,317 unique probes targeting 269,094 genomic loci, with 145,426 loci overlapping EPICv2 targets (Figure S1A). Compared to EPICv2, the MSA includes more SNP-targeting probes and a comparable number of CpH probes (Figure S1B). Human trait-associated methylations were curated from EWAS databases and literature, prioritizing statistical significance and trait diversity (STAR Methods). We broadly classified EWAS hits into 16 trait groups (Figures 1C and S1C; Table S2). As designed, the MSA is highly enriched for EWAS associations across human traits (Figure 1D), reflecting its targeted and compact design.

To target new CpGs not covered by previous Infinium platforms, we leveraged existing WGBS datasets to identify sites associated with cell type, *cis*-regulatory elements, correlation with chromatin accessibility and gene expression, 5-hydroxymethylation, and additional methylation features (Figure S1D; STAR Methods). We emphasized high-confidence cell-type-specific methylation discriminants to facilitate the deconvolution of complex heterogeneous tissue types and the study of cell-specific processes. Using pseudo bulk and sorted methylomes from brain,^49^^,^^50^^,^^51^ pan tissue,^24^ and blood cells,^52^ we performed hierarchical, non-parametric analyses to identify cell-type discriminant CpGs (STAR Methods). These analyses yielded thousands of hyper- and hypomethylated signatures across hundreds of cell types (Table S2). Despite its smaller size, the MSA includes more markers per cell type comparison than EPICv2 (Figure 1E), particularly for rarer cell types with few genome-wide designable markers. For example, our analysis of WGBS data identified 34 high-quality markers of the SRGAP1 subtype of vasoactive intestinal peptide (VIP) interneurons derived from the caudal ganglionic eminence, 31 of which were incorporated into the MSA, compared to three in EPICv2 (Figure 1E).

Like EPICv2, the MSA is highly enriched for promoter, enhancer, and transcriptionally active regions while strongly depleted from quiescent, heterochromatic, and zinc finger (ZNF) domain binding regions, as annotated by ChromHMM^53^ (Figure S1E; Table S3). Both platforms show limited representation of “open-sea” CpG sites but have a higher proportion of *cis*-regulatory element coverage (as annotated by ENCODE^54^) (Figure S1F; Table S3). Compared to EPICv2, the MSA includes a higher proportion of proximal (5.6% vs. 3.45%) and distal (16.2% vs. 10.1%) enhancer elements, with slightly reduced CpG island coverage (12.4% vs. 16.2%). The MSA’s CpH probes were selected based on brain cell-type-specific CpH methylations. These CpHs are more often linked to brain and neuron functions, implicating genes critical for neuron development and synaptic signaling (Figure 1F).

Lastly, the MSA includes at least one probe for each of 14,964 genes, defined as overlapping or within 1,500 bp of the transcription start site (TSS), nearly matching the coverage of the larger EPICv2 array (Figure S1G). The 772 genes unique to EPICv2 are enriched for olfactory receptors and highly polymorphic genes where array probe readings are often confounded by genetic polymorphism^55^ (Figure S1H). In summary, the MSA targets both human trait-associated methylations and novel, dynamic, cell-type-specific sites of biological relevance.

### MSA is highly reproducible and accurate

We used the MSA to generate 146 methylation profiles for eight cell lines (GM12878, HCT116, HeLa, Jurkat, K562, LNCaP, MCF7, and Raji) to assess the MSA’s technical performance. Most samples achieved >90% probe success rates (STAR Methods; Figure S1I). Probe detection rates were robust with 50 ng input DNA but dropped to <60% in three samples with ∼30 ng input (Figure S1I).

All cell lines showed high intra-line correlation regardless of the culturing lab, while inter-line correlations were markedly lower, reflecting differences in cell origin (Figure 1G). For GM12878 and HCT116, technical replicates from the same DNA sample yielded highly consistent methylation profiles, with F1 scores of 0.976 and Spearman’s *ρ* of 0.986 and 0.945 for GM12878 and HCT116, respectively (STAR Methods; Figure S1J). We also compared the GM12878 profiles from the MSA to those previously generated on EPIC and EPICv2 arrays using the same DNA.^44^ Correlations exceeded 0.97 across shared probes (Figure S1K), and replicate probes exhibited low measurement variance as designed (Figure S1L; STAR Methods).

Next, we evaluated the accuracy of the MSA by comparing MSA beta values with methylation titration standards. For each titration, the beta value distributions center on the expected levels (Figure 1H). We further compared MSA methylomes to those generated from the same DNA using an enzymatic methyl-sequencing (EM-seq) protocol^56^^,^^57^ (Figure 1I). We observed high intra-line, but not inter-line, correlations. Similar patterns were seen when comparing MSA data to public WGBS profiles of the same cell lines (Figure S1M). These results confirm that the MSA yields accurate methylation measurements consistent with ground-truth titrations and WGBS data.

While the MSA is more scalable than prior platforms due to its smaller size, many probes from earlier platforms were not retained (Figure S1A). We assessed whether this loss potentially limits the compatibility with prior models and associations. We noted that this loss minimally affected the performance of eight prior epigenetic clocks (Figure S1N). We also reason that missing EPIC probes can be imputed. We implemented a sparse nearest-neighbor graph approach on a deep WGBS dataset of sorted human cells^24^ with high coverage across both platforms (STAR Methods). 471,145 of the 714,492 non-retained sites had a nearest neighbor with a correlation of >0.5 across the WGBS methylomes. To evaluate model compatibility, we trained a tissue prediction model using only legacy EPIC probes and applied it to MSA-profiled human tissues. The reading at the nearest-neighbor MSA site was sufficient to predict the tissue type using the EPIC-only model (Figure 1J). A full list of nearest-neighbor mappings is provided in Table S4 for imputation use.

### MSA uncovers tissue-specific methylation biology

**Figure 2:**
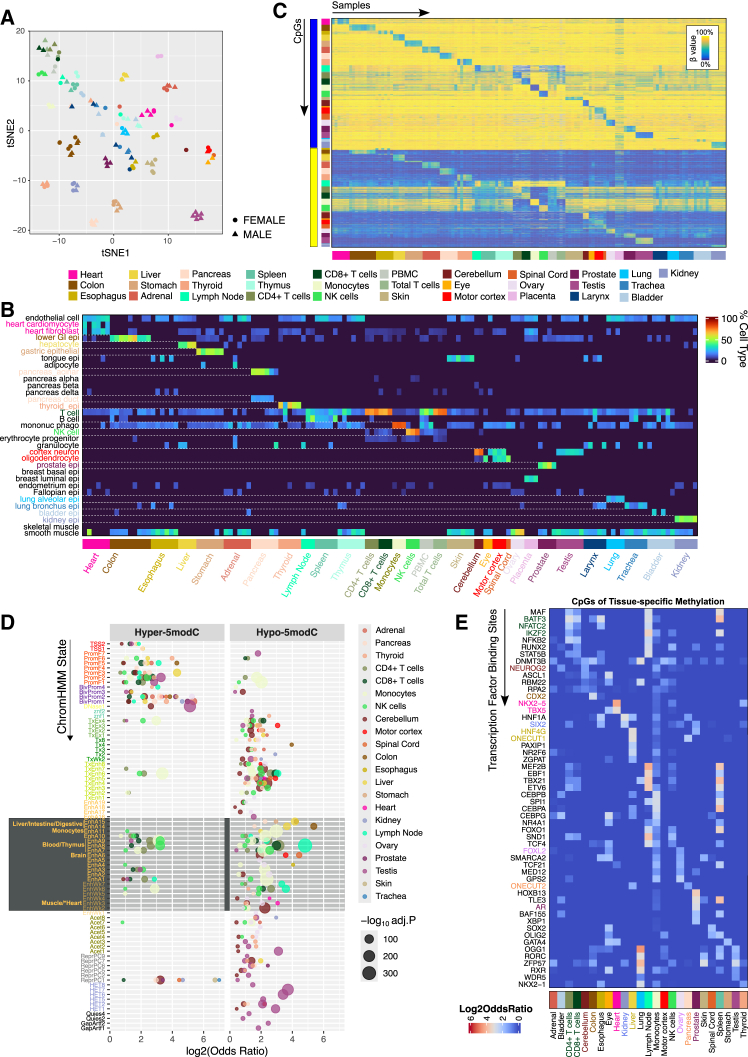
MSA reveals tissue-specific methylation biology and tissue compositions (A) t-SNE plot showing unsupervised clustering of MSA-profiled bulk tissues and sorted immune cells. (B) Heatmap of cell-type proportion estimated by methylation-based deconvolution; columns are MSA-profiled tissues, and rows are reference cell types. (C) Heatmaps of tissue-specific methylations (rows) across samples (columns). The bottom annotation bar indicates discriminated tissue; the left bars annotate hyper- vs. hypo-5modC across tissues. (D) Enrichment of hyper- (left) and hypo- (right) 5modC tissue-specific CpGs in full-stack ChromHMM states (false discovery rate [FDR] < 0.05). Circle sizes represent −log_10_(FDR-adjusted *p* values) from one-tailed Fisher’s test. (E) Heatmap showing enrichment (log_2_ odds ratio) of tissue-specific hypo-5modCs (columns) in transcription factor binding sites (rows). Row labels are colored when the transcription factor is tissue specific and enriched in the matching tissue-specific CpG sets.

We generated 18 methylomes for five different sorted immune cell types (CD4 T, CD8 T, total T cells, natural killer [NK] cells, and monocytes), peripheral blood mononuclear cells (PBMCs), and 117 methylomes from 25 different human tissue types (Figure S2A). Unsupervised t-distributed stochastic neighbor embedding (t-SNE) revealed a clear colocalization of related cell and tissue types, reflecting global methylome similarities (Figure 2A).

Cell-type proportion is a major driver of bulk tissue EWAS signals.^58^ Using reference-based deconvolution, we tested whether bulk MSA tissue methylomes could be resolved into constituent cell types (STAR Methods). Estimated cell proportions aligned well with known tissue biology (Figure 2B; Table S5). For example, heart samples were predicted to contain cardiomyocytes, heart fibroblasts, and endothelial cells, while liver samples were dominated by hepatocytes. Immune-related organs, such as the spleen and lymph nodes, showed mixed monocytes, T cells, and B cells. The thymus lacked B cells, which is consistent with its role as an organ of T cell maturation.^59^ A few samples had discordant cell proportions and did not cluster with their tissue group. For example, while most pancreatic tissues were estimated as acinar and ductal cells, the most populous cell types of the organ,^60^ one sample was enriched for granulocytes, suggesting blood contamination or sample mislabeling. Such cases were indicated and excluded from downstream tissue-specific analyses (STAR Methods).

Next, we performed one-vs.-rest non-parametric analyses to identify tissue-specific CpG discriminants (STAR Methods), revealing thousands of uniquely modified sites per tissue (Figure 2C; Table S6). Since bisulfite conversion does not distinguish 5mC from 5hmC, we refer to total modifications as “5modC” and discriminants from the standard arrays as hypo- or hyper-5modC sites. Most were hypomodified compared to other tissues (Figure S2B; Table S6). These tissue-specific probe sets were highly enriched in the cell-specific CpG signature lists curated from the analysis of public single and sorted cell datasets during array design (Figure S2C; STAR Methods), supporting both the array design and the discriminative performance of the selected probes.

To explore the role of tissue-specific methylation markers in the corresponding tissue biology, we analyzed the chromatin state distributions and gene linkages of the CpG sets. We first compared them with the full-stack ChromHMM states, a universal genome annotation learned from over 1,000 datasets comprising diverse cell types^53^ (Figure 2D). Hypermodified tissue signatures were generally absent from enhancers and were enriched in promoter and bivalent promoter states, while hypomodified markers were enriched in enhancers and gene bodies. The signatures are strongly enriched in the chromatin state associated with the matching cell type. For example, cerebellum and motor cortex signatures are enriched in EnhA6, representing brain enhancers. In contrast, colon and liver signatures were strongly enriched in EnhA14/A15, annotated as liver/digestive/intestine enhancers. The monocyte, NK cell, CD4^+^ T cell, and CD8^+^ T cell signatures were specifically enriched in EnhA7, a blood enhancer state.

In addition to tissue-specific chromatin states, the signatures colocalized with the corresponding tissue-specific transcription factor (TF) binding sites (Figure 2E). For example, CpG markers of kidney tissues were enriched in the binding sites of SIX2, which regulates the specification and maintenance of nephron progenitors,^61^ while colon signatures were enriched in the binding of CDX2, which governs intestinal development and gene expression.^62^ The markers were also in proximity to tissue-specific genes. We linked each tissue CpG marker to all genes within 10 kb and co-embedded the linked gene sets with the human gene atlas ontology database (Figure S2D). Related tissue types are localized in the gene set network, and ontology terms match the tissue type. Collectively, our MSA data uncovered the epigenome signatures at tissue-specific TF binding sites and genes that regulate the corresponding tissue biology.

Lastly, we analyzed the mitotic histories of the different tissue methylomes using a subset of Polycomb-targeted CpGs^63^ and partially methylated domains (PMDs) to track the cumulative cell divisions of the tissue (Figure S2E). Applying the models to our tissue and immune cell methylomes yielded division rates consistent with the relative proliferative activity of these tissues reported in the literature based on radioisotope labeling.^64^ For example, the colon, small intestine, and T cells had the highest division rate score, consistent with the high cellular turnover of these tissues (Figure S2E). In contrast, tissues with higher fractions of post-mitotic cell types, such as the motor cortex, cerebellum, and kidney, had the lowest division rates. Mitotic activity estimates from PMD methylation largely correlated with those from Polycomb-targeted sites. Interestingly, pancreatic and adrenal tissues showed lower PMD methylation despite similar Polycomb target-based predictions. These effects were not fully explained by global methylation differences, which were minor for tissues of similar mitotic activity based on the EpiTOC2 model (Figure S2F). The physiological cause or consequence of this PMD hypomethylation in pancreatic tissue biology warrants further investigation.

### MSA reveals 5mC-5hmC interplay across chromatin contexts

**Figure 3:**
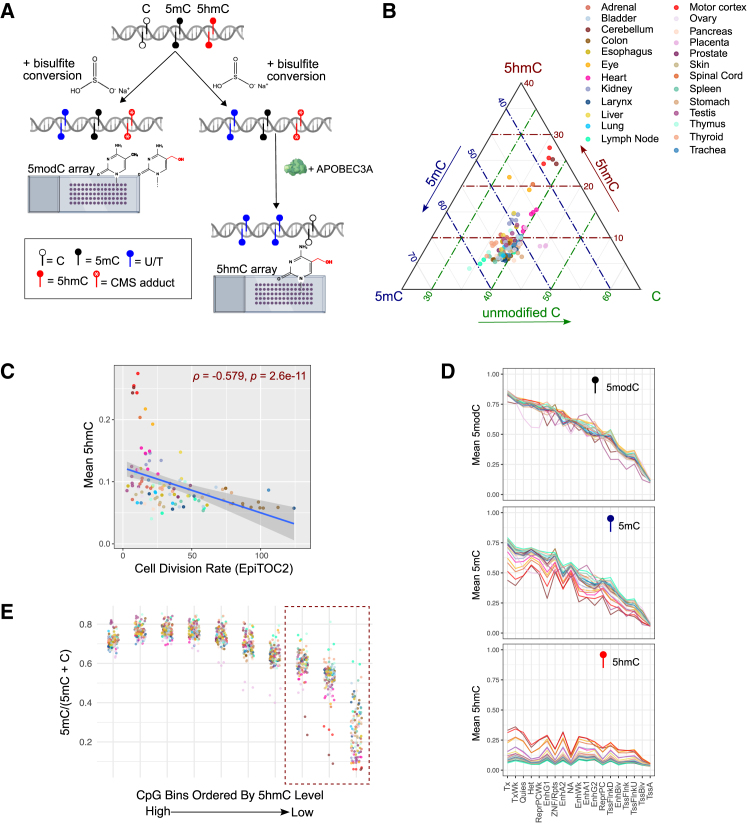
Analysis of global 5hmC across human tissues with MSA (A) Schematic of bisulfite-based 5modC (left) vs. direct 5hmC profiling (right), where APOBEC3A selectively deaminates 5mC but not cytosine-5-methylenesulfonate (CMS) adduct. (B) Ternary plot showing global levels of unmodified cytosine (bottom), 5-mC (left), and 5-hmC (right) across tissues. (C) Spearman correlation of cell division rate computed with EpiTOC2 (*x* axis) with mean global 5hmC levels across tissues (*y* axis). (D) Mean 5modC (top), 5mC (middle), and 5hmC (bottom) across consensus ChromHMM states, averaged by tissue type. (E) Scatterplot of the average 5mC/(5mC + unmodified C) ratio vs. binned 5hmC levels across tissues (*x* axis).

The standard array preparation based on bisulfite conversion does not discriminate 5mC from 5hmC.^65^ To test if the MSA is compatible with 5mC-5hmC co-profiling, hence producing a *ternary code* (5mC, 5hmC, and unmodified C) methylome, we employed a bACE-seq protocol that produces both the total modification and 5hmC profiles using two matched array experiments^66^ (Figure 3A; STAR Methods). The 5hmC profiling is based on further deaminating 5mCs using APOBEC3A, while bisulfite-converted 5hmCs resist further deamination. We produced matched 5hmC profiles of the same 117 tissue samples above. 5mC can be indirectly quantified by subtracting 5hmC measurements from the total 5modC levels obtained on matched samples. To validate 5hmC measurements, we compared probe sets designed for tissue-specific 5hmCs identified from published 5hmC-Seal^39^ and hmC-CATCH^38^ datasets. While brain tissues had high 5hmC levels across most design groups, the non-brain tissues had the highest 5hmC in the designed tissue groups (Figure S3A).

The derived 5hmC levels were globally anti-correlated with the proliferation rate of the tissue (Spearman’s *ρ* = −0.579, *p* = 2.6e−11), being most abundant in neuron-enriched central nervous system tissues, followed by the kidney, heart, and liver, and lowest in the colon and lymph node (Figures 3B and 3C). This is consistent with the biology that 5hmCs are not directly copied in mitosis and become diluted in proliferating cells.^67^ While many tissues were similar in global 5modC levels, decomposing 5modC into 5mC and 5hmC revealed tissue-specific patterns of each modification’s contribution (Figures 3B and 3D).

5mC and 5hmC share both similarities and differences in their genomic distributions. The two modifications are both enriched in gene bodies marked by H3K36me3 and H3K79me2 and depleted at promoters and TSSs (Figure 3D). However, 5hmC is more enriched than 5mC at enhancers, whereas 5mC is more elevated than 5hmC in heterochromatin and repeat regions. To better understand the biochemical relationship of 5mC and 5hmC, we quantified 5mC levels at CpG sites of varying amounts of 5hmC. We found that as 5hmC increased, the 5mC/(5mC + C) ratio also rose (Figure 3E), supporting the notion that 5mC serves as a substrate for 5hmC generation. Together, these analyses show that 5mC and 5hmC occupy overlapping but distinct chromatin compartments, with 5hmC accumulation dependent on cell proliferation rates and 5mC presence.

### MSA reveals the role of 5hmC in human tissue identity definition and biology

**Figure 4:**
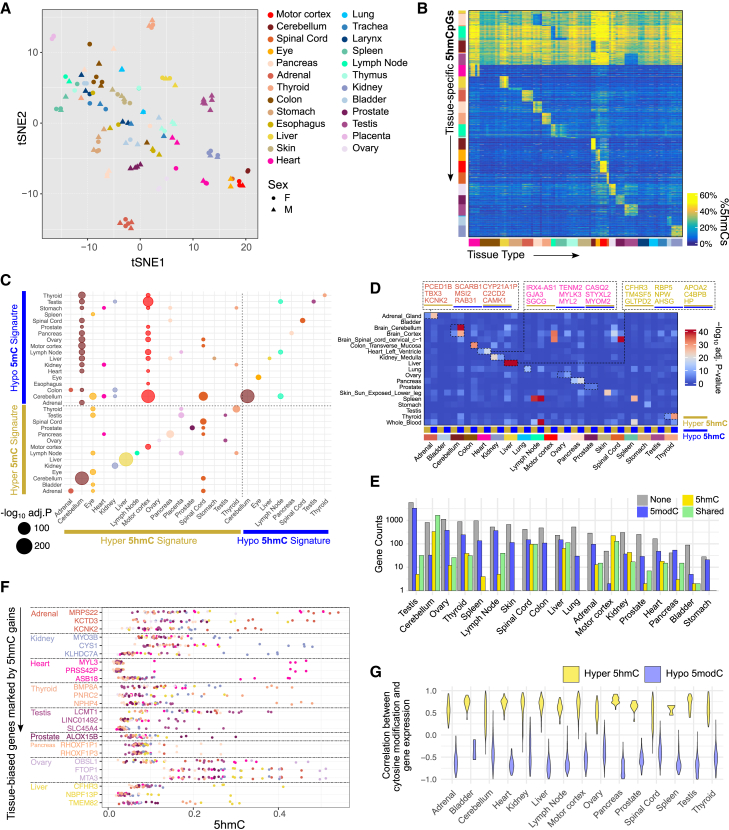
MSA reveals the role of 5hmC in human tissue identity definition (A) t-SNE plot of bulk tissues clustered by 5hmC profiles. (B) Heatmap of representative one-vs.-rest 5hmC tissue signatures (rows) across tissues (columns). The annotation bars are colored by tissue type. (C) Dot plot showing enrichment of 5modC tissue signatures in 5hmC signatures. Circle sizes represent −log_10_(FDR-adjusted *p* values) from one-tailed Fisher’s test. (D) Heatmap showing enrichment of genes linked to hyper-5hmC and hypo-5modC CpGs (columns) in tissue-specific expression gene sets identified using GTEx data of matched tissue types (rows). The annotation bar represents whether the query gene set is hyper-5hmC linked (yellow), hypo-5hmC linked (blue), or both. A one-tailed Fisher’s test for enrichment was used. (E) Grouped bar chart showing the number of tissue-specific genes marked by no tissue-specific cytosine modifications (gray), hypo-5modC (blue), hyper-5hmC (yellow), and both modifications (green) for each tissue type. (F) Tissue-biased genes marked only by tissue-specific 5hmC. The *x* axis shows mean 5hmC over linked probes; genes are colored by tissue specificity. (G) Violin plots of Pearson correlations between tissue-specific methylations and expression of linked genes, grouped by tissue and the two types of modification changes.

Having established the global pattern of 5hmCs and their interaction with 5mCs, we next asked whether 5hmCs also define tissue identity like 5modCs and the role tissue-specific 5hmCs play in development and tissue biology. First, global analysis suggests that tissue type predominates 5hmC profile similarities (Figure 4A). We performed discriminant analysis to identify thousands of tissue-specific 5hmC sites (Figure 4B; Table S7; STAR Methods). The majority of the tissue specificities were characterized by elevated 5hmCs in the target tissue compared to non-target tissues (Figure S4A). Few markers were identified for proliferative tissues, such as skin (*n* = 1) and colon (*n* = 0), consistent with their low global 5hmC levels.

As previous studies cannot discriminate 5hmC from 5mC in analyzing tissue-specific biomarkers, we use our dataset to ask if some of the 5modC markers are attributable to 5hmCs. Intriguingly, tissue-specific 5hmCs were highly enriched in the tissue-specific 5modC gains we identified from the matched tissue samples (Figure 4C) and in an independent WGBS dataset of sorted human cells^24^ (Figure S4B). 13 of 16 tissue types displayed this enrichment pattern, whereas no tissues had enriched 5hmC in tissue-specific hypo-5modC (Figure 4C). In line with these observations, the tissue-specific 5hmCs were enriched in promoters and, to a lesser extent, enhancers (Figure S4C), recapitulating the chromatin state enrichment pattern of hyper-5modC (Figure 2D). Functional enrichment analysis revealed a difference between 5modCs and 5hmCs in their genomic positions relative to TF binding. While tissue-specific TF motifs enrich tissue-specific 5modC loss, they are not preferentially found at sites with tissue-specific 5hmCs (Figure S4D).

Despite the lack of overlap between tissue-specific 5hmCs and tissue-specific TF binding sites, 5hmC still accumulated in genomic regions of tissue relevance. We derived tissue-specific gene sets using GTEx gene expression data (STAR Methods). We then tested them for enrichments of genes proximal to tissue-specific 5hmCs and 5modC loss. Genes with tissue-specific RNA expression showed strong enrichment in those marked by tissue-specific modifications (Figures 4D and 4E). For example, liver-specific 5hmC sites are associated with genes such as *APOA2*, *HP*, and *TM4SF5*, which show biased expression in the liver.^68^ Similarly, heart-specific 5hmCs are localized to *CASQ2*, *STYXL2*, and *SGCG*, which regulate sarcoplasmic reticulum functioning and heart physiology.^69^^,^^70^ For each tissue type, we quantified how many tissue-specific genes were marked by 5hmC, loss of 5modC, or both (STAR Methods; Figure 4E). This analysis revealed multiple modes of tissue identity regulation, whereby some tissue-specific genes are demarcated by the gain of 5hmCs only (Figure 4F), by the loss of 5modCs only (Figure S4E), and sometimes by both modes acting at different loci within the same gene (Figure S4F).

Next, we investigate the expression implication of tissue-specific modifications. For each CpG-gene pair, we quantified the correlation of cytosine modifications with gene expression across all tissues. For 5hmC, we found nearly exclusive positive correlations, where increasing 5hmC correlated with increasing expression levels of the gene (Figures 4G and S4G; Table S8). In contrast, tissue-specific 5modC was ubiquitously negatively correlated with the expression of the linked genes. Interestingly, the gene expression modulation by cytosine modifications may be continuous or binary (Figure S4G). Together, these two modifications appear to be complementary in regulating tissue-specific gene expression and defining cell identity.

### 5modC and 5hmC methylation biology in imprinting, aging, and sex specificities

To further explore methylation biology, we analyzed constitutive 5modC patterns across all profiled tissues. We identified 13,633 probes that were consistently unmodified (β < 0.2) and 5,012 that were consistently modified (β > 0.8) (Table S9). Constitutively modified CpGs were enriched in gene bodies, while unmethylated sites were predominantly found in CpG islands and TSSs (Figure S5A). Both categories were depleted in enhancer regions, which showed greater variability and are critical for tissue-specific regulation (Figure 2D).

**Figure 5:**
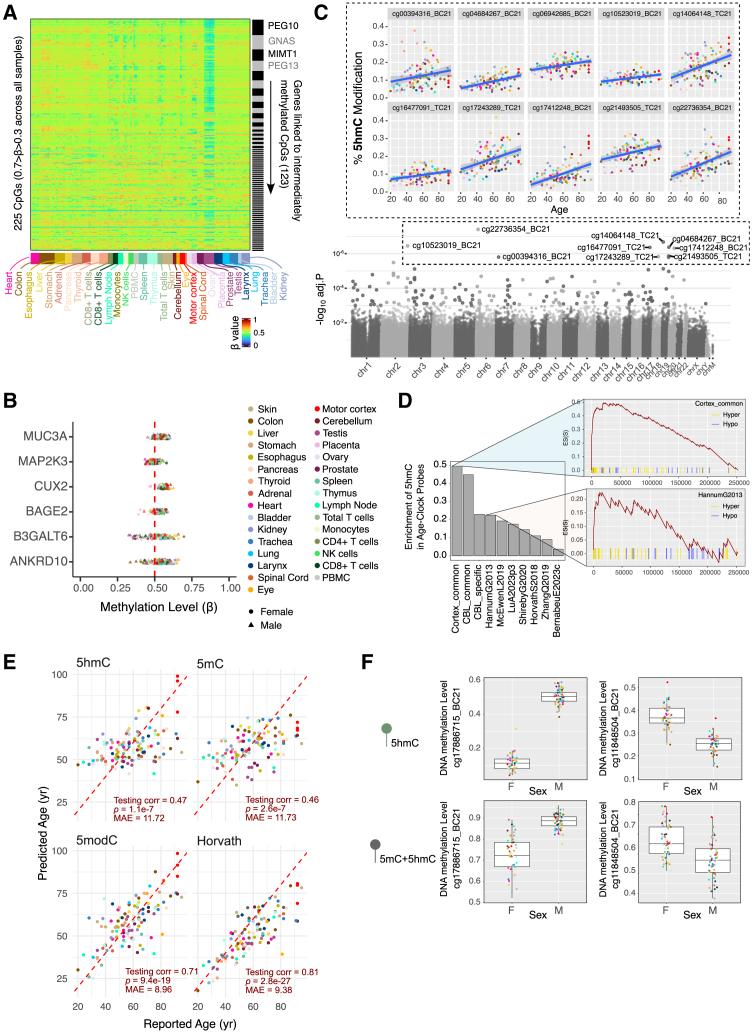
5modC and 5hmC methylation biology in imprinting, aging, and sex specificities (A) Heatmap showing beta values across all tissues (columns) for CpGs (rows) with intermediate 5modC levels (0.3–0.7), suggestive of monoallelic DNA methylation. (B) Mean beta values for intermediately modified probes for six representative genes, showing patterns resembling known imprinting genes. (C) Manhattan plot of aging 5hmC EWAS signals (bottom) and scatterplots for representative age-associated 5hmC CpGs (top). (D) Enrichment of 10 epigenetic clocks in age-associated 5hmC probes. Probes are ranked according to the *p* value of the 5hmC-age association. Representative clocks are shown on the right. (E) Age prediction using clocks trained on 5hmC (top left), 5mC (top right), 5modC (bottom left), and Horvath clock (bottom right); *x* axis: self-reported age, *y* axis: predicted age. Plots show Pearson correlation coefficients, *p* value, and mean absolute error of predictions. (F) Boxplots of 5modC and 5hmC beta values at representative autosomal CpGs with sex-specific methylation.

A total of 225 CpGs displayed intermediate methylation across all samples (β between 0.3 and 0.7), with the testis deviating most from 0.5 due to the presence of haploid spermatocytes (Figure 5A; Table S9). We linked intermediately methylated probes to nearby genes within 5 kbp, identifying 123 proximal genes. Notable linked genes included known imprinting loci such as *PEG10*, *GNAS*, and *MIMT1*, which exhibit parent-of-origin-specific expression regulated by DNA methylation at imprinting control regions (ICRs) and differentially methylated regions (DMRs) (Figure S5B). Other linked genes displayed consistent intermediate methylations but are not documented as imprinted or monoallelically expressed (Figure 5B).

Next, we analyzed age-associated patterns using linear regression, identifying widespread modification changes, the majority of which showed age-related gains (Figure S5C; Table S10; STAR Methods). Figure 5C highlights CpGs that exhibit tissue-independent 5hmC accumulation during aging. CpGs with age-associated 5hmC gains were significantly enriched in CpG islands, TSSs, and PRC2 target regions (Figure S5D, left). The similarity between 5hmC and 5modC dynamics during aging^71^ (Figure S5D, right), along with significant overlap between 5modC and 5hmC aging CpGs identified by set enrichment analysis (Figure S5E), suggests that some age-related hypermethylation may, in part, reflect 5hmC accumulation.

Given that hyper-5hmCs contribute to hyper-5modC during aging, we further ask if epigenetic clocks trained on 5modC data might have leveraged age-associated 5hmC gains in predicting chronological and biological ages. We assessed 20 epigenetic clocks and found significant enrichment of clock probe sets in 5hmC aging probes, implying that clocks incorporate, to different degrees, 5hmC to estimate age (Figure 5D; STAR Methods). In fact, 5hmC profiles alone can predict chronological age in our dataset with accuracy comparable to predictions made based on 5mC profiles (Figure 5E; Table S11). Comparatively, 5modC data generated the best aging prediction models and performed similarly to the established Horvath clock. This is likely due to 5modC clocks using both 5mC and 5hmC aging patterns. Figure S5F illustrates such a representative probe that may contribute to a 5hmC and a 5modC clock but not a 5mC clock. Further investigation is needed to determine if a deviation of 5hmC age from chronological age is reflective of biology.

Lastly, thousands of CpG sites showed sex-associated 5mC and 5hmC patterns, with 1,809 sites shared between the two modifications (Figures S5G and S5H; Table S12). Most sex-associated 5modCs are linked to X chromosome inactivation and enriched at CpG islands and TSSs on the X chromosome (Figure S5I). We also identified 966 autosomal CpGs with sex-associated 5mC and 79 with sex-associated 5hmC, some showing differences as pronounced as those seen at X-linked CpGs (Figure 5F). The mechanisms underlying sex-specific methylation at autosomal loci and its potential role in regulating sex-specific expression and phenotypes remain to be explored.

### MSA methylomes reveal strong tissue contexts of human trait associations

**Figure 6:**
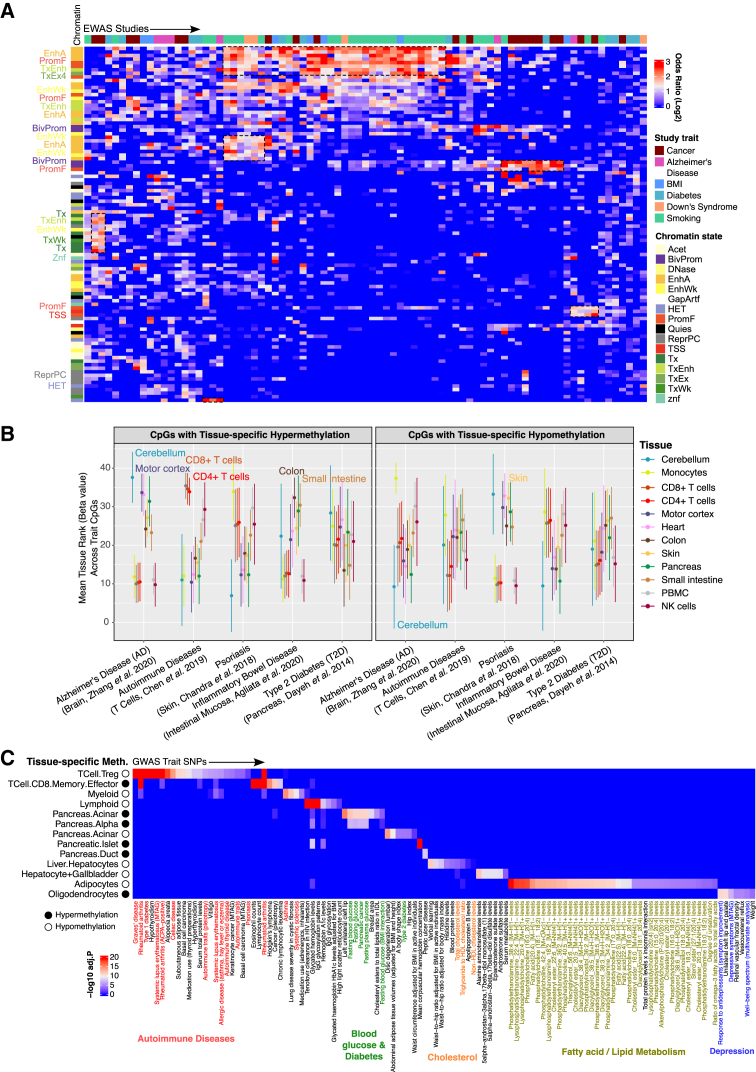
Tissue context of human trait associations (A) Heatmap of enrichment (log_2_ odds ratio) for trait-associated probes across chromatin states; columns are studies, and rows are chromatin states. (B) Distributions of tissue mean beta value ranks across trait-associated CpGs. (C) Enrichment of MSA tissue-specific methylation sets in various traits’ GWAS SNPs (one-tailed Fisher’s test, color indicates −log_10_[FDR-adjusted *p* value]).

Leveraging the trait association focus of the MSA, we evaluated the capacity of MSA data to perform context annotation of EWAS hits. In this analysis, we focused on the tissue context using the primary tissue profiles produced in this study. We first note that for the traits investigated in the curated studies, trait-associated probes are more often significantly enriched in enhancers and promoters^53^ but underrepresented in heterochromatic and repressive genomes (Figure S6A), consistent with their roles in transcriptional regulation. Traits characterized by genomic alterations (e.g., Down syndrome), cell proliferation (e.g., malignancy), and frequent toxin exposure (e.g., smoking) had distinct and recurring chromatin feature enrichment (Figure 6A). In contrast, complex disease traits, e.g., diabetes and Alzheimer’s disease, are varied in chromatin state enrichment across studies.

As expected, the enhancer- and promoter-associated probes are more variably methylated across primary human tissue types (Figure S6B). To test whether such variation reveals the tissue context of each trait, we grouped CpGs by their associated traits and compared the methylation levels across tissue types (Figure 6B; Table S13). An intriguing correspondence between the perceived tissue context and the methylation rank emerged. For example, CpGs associated with Alzheimer’s disease showed the most extreme methylation in brain tissues compared to other tissue types (Figure 6B). Sites with disease-related methylation gains have the greatest methylation readings in the brain, whereas sites with reduced methylation in diseases were least methylated in brain tissues. Similarly, probes associated with inflammatory bowel disease (IBD) were most methylated in the colon and small intestinal tissues. These results suggest a propensity of trait-associated CpGs to colocalize with differential methylations specific to the tissue that manifests the trait phenotype, underscoring the importance of tissue context when conducting EWASs.

We also investigated the extent to which genome-wide association study (GWAS) variants colocalize with tissue-specific methylation. We tested the enrichment of trait-associated SNPs in the one-vs.-rest cell-specific methylation signatures identified above (STAR Methods). These analyses identified multiple genetic variants associated with a tissue-specific trait colocalizing with the methylation signature of the corresponding tissue type. For example, SNPs associated with blood glucose and diabetes were colocalized with methylation markers for pancreatic cell types, while cholesterol variants were localized to hepatocyte-specific methylations (Figure 6C). Diverse autoimmune disorders were enriched in CpG markers for regulatory T cells, which are involved in immune system homeostasis and autoimmune suppression.^72^ Whether the genetic variants implicated in these diseases directly impact nearby tissue-specific methylation to perturb gene expression and function requires follow-up studies.

### MSA detects inter-individual methylation variation at EWAS trait sites

To date, thousands of traits have been analyzed in EWASs using peripheral whole blood, a clinically accessible tissue source that provides sufficient DNA for array-based analysis. To explore immune cell dynamics and evaluate the array’s capacity for detecting inter-individual variation, we analyzed 64 whole-blood samples from anonymous donors using the MSA. The MSA design included some major epigenetic clocks (Figure S7A), and we verified that we could accurately predict age using the multi-tissue Horvath clock^73^ on the tissues we previously profiled (Figure S7B). The Horvath clock and a sex prediction model (STAR Methods) applied to the whole-blood samples revealed a broad age range (8.7–58.4 years) and a sex distribution of 14 females and 50 males (STAR Methods; Figure S7C).

**Figure 7:**
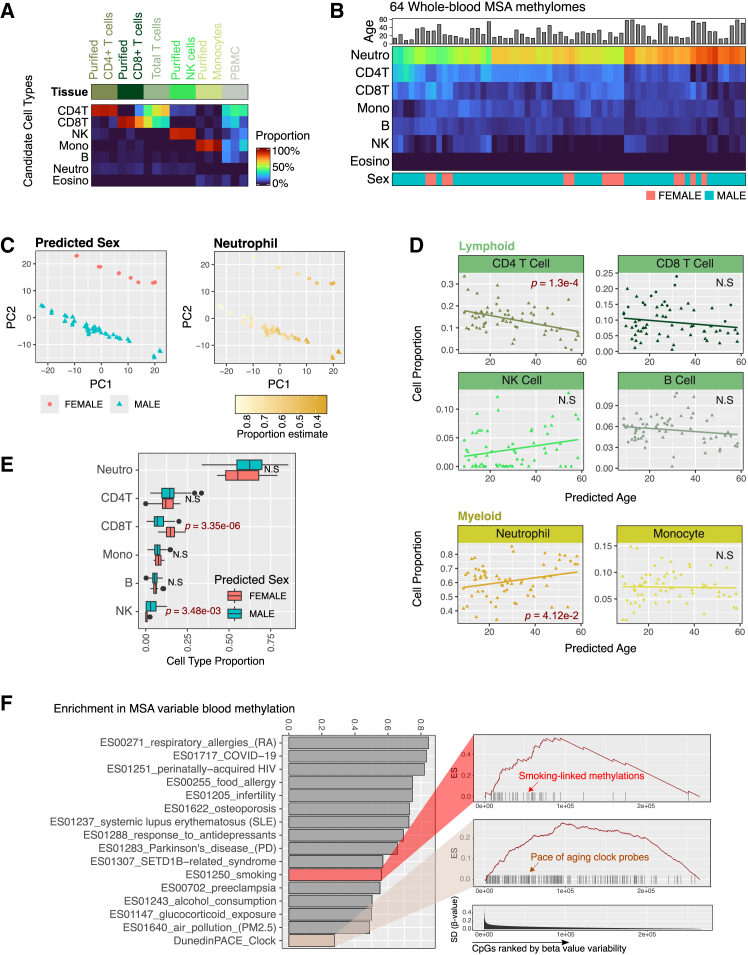
Immune cell composition and inter-individual whole-blood methylation variation (A) Validation of MSA methylome deconvolution with sorted immune cell methylation profiles; columns: MSA-profiled samples, rows: reference cell types. (B) Estimated immune cell proportions in 64 whole-blood methylomes. (C) Principal-component analysis (PCA) shows immune cell composition and sex as major sources of variance. (D) Age-related immune cell composition dynamics: CD4^+^ T cell proportions decrease and neutrophils increase with age. *p* values testing slope coefficients in linear regression are plotted. (E) Sex differences in immune cell composition. *p* values testing sex-specific slope coefficients in linear regression are plotted. (F) Enrichment of EWAS trait-associated CpGs in sites with high inter-individual methylation variation in whole-blood samples.

Cell composition explains most bulk-tissue epigenetic variations. To analyze inter-individual cell composition variation using DNA methylation, we benchmarked computational deconvolution on MSA-based methylation profiles of sorted immune cells. As expected, predicted sorted immune cells contained >90% of the matching cell type, consistent with standard purification yields (STAR Methods; Figure 7A). Then, we applied the same deconvolution strategy to whole-blood DNA methylomes. The results yielded estimates aligned with prior literature (Figure 7B; mean estimates: neutrophils, 61%; CD4 T cells, 14%; CD8 T cells, 9%; monocytes, 7%; B cells, 6%; and NK cells, 3%). Principal-component analysis showed that immune cell proportions, along with sex, explained the greatest variance in the dataset (Figures 7C and S7D). To examine immune cell composition dynamics, we regressed cell-type proportions on predicted age and sex. We found that aging was associated with a significant decrease in CD4^+^ T cells (*p* = 1.30e−4) and an increase in neutrophils (*p* = 4.12e−2) (Figure 7D). Sex differences revealed higher CD8^+^ T cell proportions (*p* = 3.35e−6) and lower NK cell proportions (*p* = 3.48e−3) in females (Figure 7E).

To further assess inter-individual variations, we ranked autosomal probes by standard deviation across individuals. Using a set enrichment framework (STAR Methods), we observed that sites with inter-individual methylation variation are significantly enriched in EWAS traits previously reported by blood-based EWASs, including immune system disorders and other environment-related traits (e.g., smoking and alcohol consumption) (Figure 7F). The new MSA probe designs showed a similar distribution of inter-individual variations compared to legacy probes, suggesting an expanded capacity for detecting blood-based methylation-trait links (Figure S7E). While we could not directly correlate methylation with phenotypic traits in our dataset, the results demonstrate that the MSA detects methylation variations associated with various physiological outcomes identified in prior studies.

## Discussion

The Infinium DNA methylation BeadChip is a broadly used and accessible assay in human population studies. It has enabled trait association discoveries and predictive models, such as epigenetic clocks, risk scores, and disease classifiers. Previous Infinium BeadChips have been designed to target genomic features, such as gene promoters, gene bodies, and *cis*-regulatory elements. While methylation variation at these genomic features is indeed associated with human traits, evenly covering genomic elements is not as economical for trait screening applications as in discovery and hypothesis generation settings.

The existing methylation-based screening of most human traits requires relatively few loci. For instance, the Horvath clock for chronological age used 353 CpGs.^73^ Other epigenetic clocks use feature numbers ranging from a few CpGs to 10,000 CpGs,^74^ which are much smaller in number than existing Infinium array capacities.^43^ The feasibility of such minimalistic approaches has also been established in cancer classification^48^ and cell-type deconvolutions,^75^ demonstrating high inference precision. The development of the MSA can be seen as a balanced approach to DNA methylome-based trait screening, prioritizing only the probe sets that link to diverse traits and high-confidence prediction models for the benefit of profiling larger human populations.

While legacy probes were incorporated for their established trait associations, the enhanced scalability of the MSA may facilitate the repositioning of these probes for novel associations. Historically, populations of European descent have been overrepresented in EWASs, potentially overlooking disease-relevant associations in more diverse demographics. Re-examining these associations in larger and more balanced cohorts will be imperative to dissecting the complex interplay of genetic and environmental influences on disease phenotypes. The legacy probe designs chosen for inclusion in the MSA are also frequently associated with multiple traits, implying that multiple physiological or environmental stimuli can converge on similar epigenetic programs. Future studies may elucidate whether these shared signatures represent common inflammatory or homeostatic pathways that are similarly disrupted and whether additional, currently under-studied disease states converge on the same loci.

Besides offering a balanced approach in trait screening, the MSA also represents an upgrade of Infinium array content to bridge deep, cell-type-resolution profiling and cost-effective population screening. While offering greater cell-type variation and genome-wide details, single-cell methylome profiling cannot be scaled to population settings. The MSA is designed to translate the cell-type-specific knowledge from single-cell and bulk whole-genome methylome profiles for use in the population setting.

Computational cell-type deconvolutions are powerful methods for interrogating tissue composition variation in development and disease. The expanded cell-specific CpG markers and refined annotation in the MSA enhance deconvolution granularity compared to EWASs based on previous Infinium platforms. For example, the commonly used cell epigenotype specific (CETS) algorithm for estimating brain cell proportions estimates NeuN+:NeuN− proportions without predicting trait-relevant subtypes.^76^ We designed cell-specific probes discriminating 174 unique cell types (82 brain cell types, 51 pan tissue, and 41 blood) and anticipate that these markers will enable high-resolution deconvolution, augmenting the study of selectively vulnerable or rare cell populations in complex diseases and tissue types. Our results and other recent work have also identified an enrichment of genetic variants associated with complex traits within cell-specific DMRs.^22^ It is not clear the extent to which methylation changes in these cell-specific DMRs may perturb the functioning of the disease-relevant cell types. We anticipate that the MSA will permit such investigations.

Previous efforts have established the compatibility of Infinium arrays with other base conversion protocols, such as Tet-assisted bisulfite conversion, to profile 5hmC modifications.^77^^,^^78^ Our analysis suggested that the new MSA is compatible with the tandem bisulfite-A3A conversion for 5hmC profiling. We applied the 5hmC profiling to neuronal and peripheral human tissues. The tissue specificity mirrors previous sequencing-based 5hmC profiles, suggesting the feasibility of using methylation arrays to implement 5hmC profiling in large sample sets. Our data also underscore the high cell-type specificity of 5hmC signals, which are often distinct but complementary to cell-specific hypo 5modC and could be additionally used to trace cell identity and tissue composition changes. Over aging and across tissues, we identified dynamic 5hmC variations that are strongly linked to tissue-specific gene expression and aging prediction models.

### Limitations of the study

As a first application, our analysis was limited in validating the trait-associated probes selected due to limited metadata availability in our study cohort. However, we found that probes associated with some traits in the literature were variably methylated in the corresponding tissue types we profiled or had a strong tissue context according to the beta value rank by tissue type (Figures 6B and 7F). We also had limited sample sizes for each tissue. While we were still able to detect some robust tissue-, sex-, and age-associated ternary-code methylations, we suspect that larger cohort sizes will enable more nuanced detection of epigenomic dynamics across human tissues and developmental and disease states. Such large cohort screening and detection of subtle methylation shifts will be better enabled by the scalability of the MSA.

We relied on publicly available RNA sequencing (RNA-seq) data to infer how 5modC and 5hmC regulate tissue-specific gene expression. Future experiments generating matched gene expression and ternary-code methylomes using the MSA will allow direct comparisons of which modifications modulate gene expression levels and under what developmental or clinical circumstances epigenomic dysregulation directly associates with gene expression. Attempting to design a consolidated array, we were also limited in the number of CpG sites we could include and, thus, genomic feature and trait coverage. As more WGBS and array-based methylomes are generated, future designs may further refine trait- and cell-type-implicated CpG sites to maximize screening and discovery power most economically.

### Conclusion

We systematically developed, benchmarked, and applied the MSA, the next iteration of the Infinium BeadChip assay, extensively consolidating trait-associated probes from prior EWASs and single-cell and bulk whole-genome methylome profiles. Our benchmark revealed the MSA as an accurate, reproducible, and scalable next-generation Infinium human methylation BeadChip targeting trait discovery in population settings. Our first application uncovered the cell-type context of human EWAS and GWAS discoveries and dynamic 5hmC association in peripheral tissues. We anticipate the MSA to be a valuable tool for methylation screening in large human populations for trait associations and broadly dissecting the cell-type-specific mechanisms of human diseases.

## Resource availability

### Lead contact

Further information and requests for resources and reagents should be directed to and will be fulfilled by the lead contact, Wanding Zhou (wanding.zhou@pennmedicine.upenn.edu).

### Materials availability

This study did not generate new reagents.

### Data and code availability

• The complete MSA manifest, design criteria, technical, human trait, and functional annotations are available at https://zwdzwd.github.io/InfiniumAnnotation.
• Informatics for MSA data preprocessing and functional analysis is available in the R/Bioconductor package *SeSAMe* (v.3.22+): https://bioconductor.org/packages/release/bioc/html/sesame.html.
• Additional code for analyses is available at https://doi.org/10.5281/zenodo.15390877.
• The generated human cell line, primary tissue 5mC and 5hmC methylome profiles (*N* = 676), and EM-seq data are available in the GEO with accessions GEO: GSE264438 and GSE267407.

## Acknowledgments

The authors thank Lynn Chen, Max Eldabbas, and Emileigh Maddox of the Human Immunology Core at the Perelman School of Medicine at the University of Pennsylvania for assistance with immune cell purification. The HIC is supported in part by 10.13039/100000002NIH P30 AI045008 and P30 CA016520 (HIC RRID: SCR_022380). The authors thank the NCI Cooperative Human Tissue Network (CHTN) for providing human tissue samples. Other investigators may have received specimens from the same tissue specimens (RRID: SCR_004446). The authors thank the NIH for funding (R35-GM146978 to W.Z. and R01-HG010646 to R.M.K.).

## Author contributions

Conceptualization, W.Z., N.R., R.P., and D.C.G.; methodology, D.C.G. and W.Z.; formal analysis, D.C.G. and W.Z.; investigation, D.C.G., C.C., E.K., S.M.L., M.H., M.K., S.G., A.P., B.B., M.S.W., and L.M.; resources, R.M.K., J.B.P., M.H., M.K., Q.Z., E.M., C.T., J.S., S.G., and A.P.; writing – original draft, D.C.G. and W.Z.; writing – review & editing, D.C.G., W.Z., N.R., R.P., and R.M.K.; funding acquisition, N.R., R.P., and W.Z.; supervision, W.Z.

## Declaration of interests

W.Z. received MSA BeadChips from Illumina, Inc., for research. B.B., S.G., A.P., M.S.W., L.M., E.M., M.K., Q.Z., C.T., M.H., J.S., R.P., and N.R. are Illumina employees. US patent application no. 63/596,091 has been submitted and covers the methods and findings discussed in this research.

## STAR★Methods

### Key resources table

**Table:** 

REAGENT or RESOURCE	SOURCE	IDENTIFIER
**Biological samples**		

Human DNA methylation calibration standards	EpigenDx	80-8060H-PREMIX
Primary human tissues	CHTN	https://chtn.cancer.gov/
HeLa	BioChain Institute	D1255811
Jurkat	Thermo Scientific	SD1121

**Critical commercial assays**		

MSA Methylation BeadChip	Illumina	20112612
QIAGEN QIAmp Mini Kit	QIAGEN	51306
NEBNext® Enzymatic Methyl-seq Kit	NEB	E7120
Twist Human Methylome Panel	Twist Bioscience	105520
EZ DNA Methylation Kit	Zymo Research	D5001

**Deposited data**		

Raw and analyzed MSA data	This paper	GEO: GSE264438
Raw and analyzed EM-seq data	This paper	GEO: GSE267407

**Experimental models: Cell lines**		

Human GM12878 cells	Coriell	RRID: CVCL_7526
Human K562 cells	ATCC	RRID: CVCL_0004, CCL-243
Human LNCaP cells	ATCC	RRID: CVCL_1379, CRL-1740
Human HCT116 cells	ATCC	RRID: CVCL_0291, CCL-247

**Software and algorithms**		

SeSAMe	Zhou et al.^23^^,^^79^	https://bioconductor.org/packages/release/bioc/html/sesame.html
BEDTools	Quinlan et al.^80^	https://github.com/arq5x/bedtools2
EpiDISH	Zheng et al.^81^	https://www.bioconductor.org/packages/release/bioc/html/EpiDISH.html
BISCUIT	Zhou et al.^82^	https://huishenlab.github.io/biscuit/
Enrichr	Kuleshov et al.^83^	https://maayanlab.cloud/Enrichr/
HOMER	Heinz et al.^84^	http://homer.ucsd.edu/homer/
KnowYourCG	Zhou lab, CHOP	https://www.bioconductor.org/packages/devel/bioc/html/knowYourCG.html
CytoMethIC	Zhou lab, CHOP	https://www.bioconductor.org/packages/release/data/experiment/html/CytoMethIC.html
Cytoscape	Shannon et al.^85^	https://cytoscape.org/
glmnet	Friedman et al.^86^	https://cran.r-project.org/web/packages/glmnet/index.html
methylclock	Pelegi-Siso et al.^87^	https://www.bioconductor.org/packages/release/bioc/html/methylclock.html
epiTOC2	Teschendorff^63^	https://doi.org/10.5281/zenodo.2632937
dnaMethyAge	Wang et al.^88^	https://github.com/yiluyucheng/dnaMethyAge

**Other**		

MSA BeadChip Manifest and Probe Annotation	This paper	http://zwdzwd.github.io/InfiniumAnnotation
Single-cell brain WGBS datasets	Tian et al.^22^; Luo et al.^51^; Luo et al.^49^	https://assets.nemoarchive.org/dat-jx4eu3g
Sorted human cell WGBS datasets	Loyfer et al.^24^	GEO: GSE186458
5hmC-Seal	Cui et al.^39^	GEO: GSE144530
5hmC-CATCH	He et al.^38^	GEO: GSE134078
EWAS Atlas	Li et al.^34^	https://ngdc.cncb.ac.cn/ewas/atlas
EWAS Catalog	Battram et al.^33^	https://www.ewascatalog.org
EPICv2 data	Kaur et al.^44^	GEO: GSE228820
ReMap, Human Transcription Factor Binding Sites	Hammal et al.^89^	https://remap.univ-amu.fr
Human tissue gene expression data	GTEx	https://www.gtexportal.org/

### Experimental model and subject details

#### Tissue dissection

117 Fresh frozen tissue samples (age 20–95, 55 Female 62 Male) were obtained from the Cooperative Human Tissue Network (CHTN), and 30-50mg of tissue were dissected on dry ice.

#### Cell line culture

GM12878, K562 (CCL-243), LNCaP (CRL-1740), and HCT116 (CCL-247) cells (Source 1) were obtained from American Type Culture Collection (ATCC, Manassas, VA, USA). 1-4 x 10ˆ6 cells were plated and cultured for 6 days with fresh media added 2–3 days. K562 cells were cultured in Iscove’s Modified Dulbecco’s Medium (30–2005, ATCC), 10% Fetal Bovine Serum (FBS) (45000-736, Gibco), and 1% penicillin/streptomycin (15140122, Gibco). LNCaP cells were cultured in Roswell Park Memorial Institute Medium (RPMI-1640) (30–2001, ATCC), 10% FBS, and 1% penicillin/streptomycin (15140122, Gibco). GM12878 cells were cultured with RPI-1640 (72400047, Invitrogen), and 15% Fetal Bovine Serum (Gibco, 45000-736), 1% GlutaMAX (Gibco, 35050061), and 1% penicillin/streptomycin (15140122, Gibco). HCT116 cells were cultured in McCoy’s 5a medium modified (ATCC,30–2007), 10% Fetal Bovine Serum (FBS) (45000-736, Gibco), and 1% penicillin/streptomycin (15140122, Gibco). All cells were maintained in a 37°C incubator with 5% CO2 and cultured at a 75 cm2 culture flask (Fisher, BD353136).

### Method details

#### DNA extraction

Genomic DNA was extracted from 30 to 70 mg of tissue or 5.0 × 10ˆ6 cells for Source 1 cell lines using commercially available QIAGEN QIAamp Mini Kits (QIAGEN, 51304), following the manufacturer’s protocol. DNA was quantified using a Qubit 4 Fluorometer (Invitrogen). For Source 2 and Source 3 cell lines, genomic DNA was purchased from BioChain Institute (HeLa - #D1255811, Raji - #D1255840, Jurkat - #D1255815, MCF7 - #D1255830, K562 - #D1255820).

#### Immune cell purification

Sorted immune cells were purified by the Human Immunology Core at the University of Pennsylvania following STEMCELL Technologies RosetteSep Enrichment Cocktail protocols (https://cdn.stemcell.com/media/files/pis/10000000545-PIS_02.pdf). PBMCs were isolated using a Lymphoprep ficol layer.

#### Methylation titration controls

10 ng of fully methylated human blood (Thermo Scientific, SD1131) and Jurkat (Thermo Scientific, SD1121) genomic DNA were amplified using the Repli-g Mini Kit (QIAGEN, 150023) according to the manufacturer’s protocol. Following quantification with a Qubit 4 Fluorometer, 500ng of unamplified and amplified DNA were combined for the 50% control. Human pre-mixed calibration standards (0,5,10,25,50,75,100%) were purchased from EpigenDx (EpigenDx 80-8060H_PreMix), and 200ng/titration was used for testing.

#### EM sequencing of cell line DNA

Genomic DNA from the GM12878, K562, and HCT116 cell lines were extracted according to the QIAGEN QIAmp Mini Kit Protocol. The three samples were then mechanically sheared to 300 base pairs using the M220 Focused-ultrasonicator (Covaris, 500295) and methylated lambda control DNA. 200ng of each sample was enzymatically converted using the NEBNext Enzymatic Methyl-seq Kit (NEB, E7120) with the manufacturer’s protocol. The samples were then indexed during PCR amplification during PCR amplification using EM-Seq index primers (NEB 7140). The indexed libraries (200 ng each) were pooled and used as input for the Twist NGS Methylation Detection System for target enrichment. A pre-hybridization solution of blockers and enhancers was created to prepare the pool for hybridization (Twist Bioscience, 104180). The DNA was hybridized with the Twist Human Methylome Panel (Twist Bioscience, 105520), and the targets were bound with streptavidin beads (Twist Bioscience, 100983), followed by a post-capture amplification. The enriched libraries were sequenced to 20× on the Illumina Novaseq 6000 PE150 platform.

#### 5hmC profiling

Using the EZ DNA Methylation Kit (Zymo Research, D5001), 500 ng of each sample was bisulfite converted and purified following the manufacturer’s protocol. The samples were then denatured with DMSO at 95°C for 5 min and snap-cooled on dry ice. The samples were deaminated using APOBEC3A (A3A) purified following previously published protocol^90^ over 2 h at 37°C. After incubation, the samples were purified using the Oligo Clean and Concentrator Kit (Zymo Research, D4060), following the manufacturer’s protocol. Two cycles of whole genome amplification were performed using 50 U of Klenow Fragment (3'→5′ exo-) (NEB, M0212M), dNTP solution mix (Bio-Rad, #1708874), and Random Primer 6 (NEB, S1230S). The samples were finally purified using AMPure XP Beads (Beckman Coulter Life Sciences, A63881).

### Quantification and statistical analysis

#### CpG probe selection

##### Probe designability

We aligned unmethylated and methylated probe sequences to the human GRCh38 genome using the BISCUIT tool suite.^82^ To identify uniquely mapping sequences, subsequences of 30,35,40 and the entire 50nt probe sequence were aligned, and only probe designs where all subsequences had mapping quality >20 for both the methylated and unmethylated allele were considered. For these 19,253,974 uniquely mapping CpGs, design scores reflecting hybridization efficiency and melting temperature were computed, and 13,891,035 CpGs with design scores >0.3 were retained. Any probe sequence that contained common SNPs (dbSNP Build 151)^91^ within 5nt of the 3′ end was removed. Sequences with more than six additional CpGs were also removed to prevent hybridization interference due to variable methylation of neighboring CpGs. 9,993,793 CpGs remained from this preprocessing (“Designable Probes”), from which all array content was subsequently selected. When possible, high-quality probes (design score ≥ 0.6) were prioritized. In the final MSA manifest, >99.9% of probe sequences are uniquely mapped with high quality. The minority of probes with lower-quality mapping can be readily identified in the standard SeSAMe^79^ preprocessing pipeline. Like the EPICv2 BeadChip, the MSA array includes replicate probe designs that target the same 122-mer genomic loci but may vary in the other design details.^44^ The replicate designs have the same prefix but alternative suffixes that describe the chemistry and target strand specifications.^55^ For each of the 8,523 replicate probe groups, the standard deviation (SD) of replicate probes within cell line samples was calculated and compared to the SDs of non-replicate probes to assess replicate probe measurement variance (Figure S1L). Replicate probes had a low mean standard deviation of 0.02 compared to non-replicate probes, suggesting that the replicate probes produce consistent methylation measurements. Methylation can be averaged over replicate probes or the most robust replicate selected based on signal intensity *p*-value using *SeSAMe*.^79^

##### *Cis*-regulatory elements

Human GRCh38 candidate *cis*-regulatory element (CRE) annotations were downloaded from the ENCODE Project Consortium^92^ and intersected with designable CpG sites. The methylation range for each CpG was computed across sorted immune^52^ and pan tissue^24^ cell types. CpGs that did not show a range >0.4 were filtered out. The remaining CpGs were grouped by CRE type and sorted by methylation range. 30,000 CpGs total were sampled with a bias toward enhancer elements (dELS: 64%; pELS: 21%; CTCF Only, CTCF-bound:11%; PLS:2%; DNAse-H3K4me3:2%).

##### Monoallelic/intermediate methylation

180 bulk adult normal WGBS samples (Table S1) were analyzed to identify candidate monoallelically methylated CpG sites. Autosomal CpGs with minimum coverage of 20 reads and mean methylation >0.3 and <0.7 across 140 of the 180 samples were considered intermediate methylation and intersected with the designable probe list. 207 pan-tissue sorted cell WGBS methylomes from Loyfer et al.^24^ were also analyzed for intermediate methylation, and designable CpGs with mean methylation >0.3 and <0.7 across 180 of the 207 samples were selected.

##### XCI-linked CpGs

76 high coverage (>20 million CpGs) normal female WGBS samples (Table S1) were analyzed to identify X chromosome CpG sites with intermediate methylation across samples (0.3 < methylation <0.7). An additional 95 normal male WGBS samples were analyzed to identify X chromosome CpG sites fully unmethylated (<0.3 methylation across 50 samples) or fully methylated (>0.7). The CpG sites intermediately methylated in female samples but unmethylated or fully methylated in male samples were intersected with the high-quality probe list.

##### Cell type-specific methylation

BED/bigWig files for single cell brain,^49^^,^^50^^,^^51^ sorted pan tissue,^24^ and sorted immune cell WGBS data^52^ were downloaded and used for marker identification. To reduce the sparsity of single-cell brain data, pseudo bulk methylomes were generated by averaging methylation over the cell type labels obtained by unsupervised clustering analysis previously reported. One vs. all comparisons were performed across major cell type groups and hierarchically within major groups to identify subtype markers. Wilcoxon rank sum testing was performed between the target and out groups at each CpG site to identify cell-specific markers. Designable CpG sites with an AUC = 1 and a delta beta ≥ 0.3 between the in and out groups were selected, and markers were capped at 80 CpGs per cell type contrast. Hyper and hypomethylated signatures were balanced when possible.

##### 5hmC analysis

5hmC-Seal^39^ and hmC-CATCH^38^ 5hmC peaks were downloaded (5hmC Seal – GSE144530, 5hmC CATCH - GSE134078). Genomic intervals were intersected with the designable CpG list. For 5hmC-Seal data, the 5hmC CpG signal was treated as a binary value (1 if within a significant peak, 0 if not). For hmc-CATCH data, the peak coverage was applied to CpGs within the peak, and samples were scaled according to the total coverage. Tissue-specific 5hmC sites were identified as previously described for the WGBS data. To identify 5hmC sites along a continuum of tissue specificity, the top 10K most highly covered CpGs in each sample from the hmC-CATCH data^38^ were collected and binned according to the frequencies the CpG was in the top 10K across the 60 samples. 11 bins of 5 tissue count intervals (e.g., 1–5, 6–10, …, 55–60 tissues) were sampled equally, with sampling capped at 200 CpGs per bin.

##### Cell-specific CpH methylation

Genes with cell-specific mCH methylation were downloaded,^49^ and the top ten genes with the highest AUROC were selected for each cell type. Gene coordinates were intersected with CAC cytosines, the most prevalent mCH context found in neurons. 20 cytosines were sampled from each gene for each cell type.

##### DNA methylation-gene expression correlations

Matched WGBS/Gene expression data from the Roadmap Epigenomics Mapping Consortium were used to compute the Spearman correlation between CpGs in the high-quality designability list and genes within 10KB of the CpG. CpGs were then ranked by the *p*-value of the correlation, standard deviation and expression levels of the gene, and absolute value of the correlation. The top 2,500 CpGs negatively correlated with the expression of the linked gene, and the top 2,500 positively correlated CpGs were selected. TCGA normal tissues^93^ were also analyzed to identify correlated linked CpG-Gene pairs. CpGs with a correlation ≥ 0.6 or ≤ −0.7 and a *p*-value <0.05 were additionally included (901 positively correlated, 1,620 negatively correlated).

##### DNA methylation-chromatin accessibility correlations

Matched DNA-chromatin accessibility data were downloaded from Luo et al. 2022,^49^ and Spearman correlations were computed between the accessibility peaks and CpG methylation sites. Correlations with *p*-values <0.05 and |Spearman’s *ρ*| > 0.5 were selected, and the CpGs intersected with the high-quality designability list.

##### CoRSIVs

Genomic coordinates for CoRSIVs were downloaded^94^^,^^95^ and intersected with high-quality designable probes.

##### Solo-WCGW in partially methylated domains

CpGs in the WCGW context (flanked by A or T) in common PMDs were downloaded from Zhou et al. 2018^23^ and intersected with high-quality designable probes. This subset was further intersected with CpG islands, and 6,000 probes were randomly sampled.

##### meQTLs

meQTL data was downloaded from the GoDMC database,^30^ and CpGs were ranked according to the number of times a CpG was associated with a meQTL. The top 10K CpGs were selected. An additional 20K meQTLs were randomly sampled from Hawe et al. 2021.^29^

##### Imprinting-associated DMRs

Differentially methylated regions associated with monoallelically expressed genes were downloaded from Skaar et al. 2012^96^ and lifted to GRCh38 coordinates. The DMRs were intersected with the designable probes list.

##### Y-linked genes

180 high coverage (>20 million CpGs) human WGBS samples (Table S1) were analyzed to identify variably methylated Y-linked genes. The Y chromosome CpGs were intersected with designable probes and subsequently intersected with all Y chromosome genes (GENCODE V39). The variance across the 180 samples was computed at every remaining CpG site. For each gene, the top 20 most variable probes were selected.

##### Human trait associations

1,067 EWAS studies were curated from the literature and EWAS databases (EWAS catalog,^33^ EWAS atlas^34^). A subset of these studies was manually prioritized for probe selection based on study design and results (large sample number, statistical rigor/adequate covariates in analysis, statistical significance of associated probes), diversity of trait coverage, citation number, and the journal impact factor. Of these studies, we included all designable probes or capped the selection at the top 2500 most significant probes based on *p*-value association with the trait. For the remaining studies in the databases and curation, we selected the top 100 most significant probes based on the *p*-value. Study titles and trait annotations were queried for regular expressions to consolidate all selected studies/traits into 16 major trait groups.

#### Data preprocessing and statistical analysis

All data preprocessing was done using the *SeSAMe* R package (version 1.22.0).^79^ A manifest address file was generated using the MSA manifest available at https://github.com/zhou-lab/InfiniumAnnotationV1/raw/main/Anno/MSA/MSA.hg38.manifest.tsv.gz and the *sesameAnno_buildAddressFile* function. Beta values were extracted from raw IDAT files using the *openSesame* function with the built address file and default parameters. Probe detection rates were obtained using the *probeSuccessRate* argument with the *openSesame* function. One sample with probe detection rates <0.7 was excluded from analyses. All analyses were performed using R version 4.4. The FDR method was used to adjust P-values for multiple testing corrections.

#### Trait enrichment testing

2,398,372 EWAS hits were curated from the literature and EWAS databases^33^^,^^34^ and used as a background for enrichment testing. Traits were annotated to 16 major trait groups by searching for regular expression terms relevant to the trait group within the study or trait descriptions. The odds ratio enrichment in these trait groups was computed for 3 query sets: 1) EPICv2 probes, retained MSA probes from prior Infinium platforms, and a random set of probes equal in size to the retained MSA probes. The log2 odds ratio was plotted for each platform across trait groups. For testing the enrichment of MSA and EPICv2 probes in total trait-associated probes, all EWAS probes were rank-ordered according to how many traits the probes were associated with. The MSA and EPICv2 probes were each tested as a query against the ranked probe list using a modified gene set enrichment approach^97^ using the *knowYourCG* R package (version 1.0.0).

#### Gene linkage and ontology analysis

The Infinium MSA and EPICv2 BeadChip manifests were downloaded (https://github.com/zhou-lab/InfiniumAnnotationV1/raw/main/Anno/), and probe coordinates expanded 1500bp upstream of the probe start site. The manifests were then intersected with GENCODE.v41 GTF files to identify linked genes. Gene ontology testing was performed for protein-coding genes using Enrichr^98^ which uses a hypergeometric test for enrichment. The GO Biological Process gene set was queried. For CpH probe-linked genes, only genes with a minimum of 2 probes per gene were analyzed.

#### Sample reproducibility and accuracy

Pearson correlation coefficients were computed across cell line samples (*n* = 146). Correlation matrices were plotted in heatmaps. For pairwise replicate comparisons, beta values were first binarized as 1 if beta >0.5 and 0 if beta <0.5. F1 scores for the binarized vectors were computed using the MLmetrics package (1.1.3). Tissue samples were clustered by 5hmC or 5modC methylomes using the Rtsne R package (Version 0.17) using the *Rtsne* function and a perplexity of 12.

#### Cell deconvolution

Reference-based cellular deconvolution for sorted immune cells and whole blood samples was performed using the EpiDISH R package^99^ (version 2.18.0) with the robust partial correlations (RPC) method. The centDHSbloodDMC.m matrix provided within the package was used as a reference for sorted immune cell deconvolution. For bulk tissue cell type inference, a reference for one vs. all cell-specific CpGs was created from Loyfer et al. 2023^24^ as previously described and deposited to the CytoMethIC github repository (https://github.com/zhou-lab/CytoMethIC_models/). Cell proportion scores were computed with the *cmi_predict* function from the CytoMethIC package (Version 1.1.1).

#### Identification of tissue-specific markers

One-vs-rest tissue type comparisons were performed for sorted immune cells and bulk tissues. Wilcoxon rank sum testing between the target and out-group was performed at each CpG site. CpGs with NA values in >10% of the target group or >50% of the out-group were excluded. The AUC for discriminating between the target and the out-groups was computed. Only CpGs with a delta beta >20% and AUC ≥ 0.8 were selected as cell markers for 5modC analysis (Figures 2 and S2). For visualization, the top 50 hypo and hyper-5modC CpGs sorted by AUC and delta beta were selected for each tissue type. For 5hmC signatures and comparing the numbers and genomic distributions of 5modC vs. 5hmC signatures (Figures 4 and S4), the same analysis was performed with a delta beta of >5% used as a threshold for marker identification.

#### Transcription factor binding site analysis

BED files containing TFBS peaks were downloaded from ReMap 2022 (https://remap.univ-amu.fr^97^). The peaks for each transcription factor were intersected with all MSA CpGs to create CpG-TFBS links. Tissue signatures were tested for enrichment in the TFBS CpG sets by computing the log2 odds ratio of the overlap.

#### Transcription factor motif analysis of 5modC and 5hmC tissue signatures

Tissue-specific hyper-5hmC and hypo-5modC signature probes for each tissue type were converted to BED files using the probe coordinates on the MSA manifest. Motif enrichment was tested using *HOMER* (v5.1) using the *findMotifsGenome.pl* function and the hg38 (v7.0) human genome annotation provided by the software. The top 10 most significantly enriched motifs based on q-value (Benjamini, minimum <0.05) were plotted for each tissue and signature type.

#### Enrichment testing in chromatin states

Enrichment testing in chromatin states for all probe sets in this manuscript was performed using the *knowYourCG* R package (version 1.0.0) with the consensus and full-stack chromHMM knowledgebase sets and the *testEnrichment* function (hypergeometric distribution, one-sided enrichment). All MSA probes were used as the background.

#### Tissue-specific CpG marker validation enrichment testing

BED/bigWig files for single cell brain,^49^^,^^50^^,^^51^ sorted pan tissue,^24^ and sorted immune cell WGBS data^52^ were downloaded and used for marker identification. To reduce the sparsity of single-cell brain data, pseudo bulk methylomes were generated by averaging methylation over the cell type labels obtained by unsupervised clustering analysis previously reported. One vs. all comparisons were performed across major cell type groups and hierarchically within major groups to identify subtype markers. Wilcoxon rank sum testing was performed between the target and out groups at each CpG site to identify cell-specific markers. CpG sites with an AUC >0.95 and a difference in beta value >0.5 between the in and out groups were selected to generate marker lists for each cell type and intersected with MSA probes. The 5modC and 5hmC tissue signatures identified from MSA profiled tissues were tested for enrichment in the marker lists using Fisher’s exact test with all MSA probes as the background.

#### Nearest neighbor analysis

Nearest neighbor analysis was performed using deep WGBS data^24^ to identify neighbor genomic coordinates on MSA for non-retained EPIC probes. The WGBS data was subset for the MSA probe genomic coordinates and reference graphs were constructed using the *nnd_knn (k=50 neighbors)* function from the *rnn_descent* R package (version 0.1.6). The graph was then queried using the EPIC probe genomic coordinates from the WGBS data using the *graph_knn_query* function. For each CpG, the neighbor in the reference graph with the lowest Euclidean distance was recorded. We additionally computed the Euclidean distance between every EPIC probe and the nearest genomic neighbor on MSA. The final CpG with the lowest Euclidean distance was retained. To test the performance of neighbor probes in classifying tissue type, we used an EPIC tissue prediction model from the *CytoMethIC* R package (version 1.1.1) and removed all probes from the model that were retained on MSA. For the remaining EPIC-only probes, we substituted the neighbor beta values from the MSA methylomes to compute the tissue inference.

#### Tissue-marker gene enrichment testing

5mod CpG signatures for each tissue type were linked to genes +/− 10KB from the CpG site (GENCODE V19). The resulting gene sets for each tissue type were tested for enrichment against the HumanGeneAtlas^100^ downloaded from Enrichr,^83^^,^^101^ and the top 5 most enriched ontology terms (FDR <0.05) for each tissue type’s gene sets were selected for network graphing in Cytoscape version 3.9.1 using the log2 odds ratio for edge weights and an edge-weighted spring embedded layout.

#### Generation of tissue-specific gene sets

Median gene-level TPM by tissue expression data were downloaded from the GTEx Portal (v10 RNASeQCv2.4.2). One vs. all comparisons were performed for each tissue type to identify tissue-biased gene sets. Wilcoxon rank sum testing was performed between the in-group and all other tissues at each gene to identify genes with tissue-biased expression. For each tissue type, genes with an AUC >0.8 and a delta TPM >1 were selected.

#### Tissue-specific CpG-Gene correlation analysis

Hypo-5modC and hyper-5hmC tissue signature CpGs were linked to genes +/− 50 KB using GENCODE V19 gene annotations. The resulting CpG-gene pair lists were filtered for those containing tissue-specific genes (previously described in the generation of tissue-specific gene sets). For each CpG-Gene pair, the Pearson correlation was computed.

#### Tissue-specific CpG-marker gene enrichment

Hypo-5modC and hyper-5hmC tissue signature CpGs (delta beta >0.05) were linked to genes +/− 50 KB using GENCODE V19 gene annotations. The resulting gene sets for each tissue and modification type were tested for enrichment in the derived matching tissue-specific GTEx gene sets using Fisher’s exact test.

#### Chromatin state analysis of tissue-specific CpGs

Hyper and hypo-5modC tissue signatures (delta beta >0.2) were tested for enrichment in full-stack ChromHMM states as previously described (Figure 2D). To directly compare the genomic distribution of hypo-5modC and hyper-5hmC signatures, CpG markers with a delta beta >0.05 for both modifications were tested (Figure S4C). CNS and placenta tissues were omitted due to global differences across chromatin states.

#### Epigenetic clock analysis

730 TCGA normal tissues profiled on the HM450 array were used to assess the impact of missing probes on epigenetic clock estimation. The full clock probes and the subset represented on MSA were both tested, and the predictions were compared (Figure S1N). For MSA-profiled tissues, the probe suffixes were removed, and duplicate probes were averaged. All age estimates were computed with the *DNAmAge* function from the *methylclock* package (version 1.8.0)^87^ using default parameters. HypoClock and EpiTOC2 mitotic rate estimates were computed by tissue type group using the data and code provided by the authors at https://zenodo.org/records/2632938. Placental tissues were excluded.

#### Sex prediction

Sex for anonymous whole blood donors was inferred using the *cmi_predict* function from the *CytoMethIC* R package (version 1.1.1) using the sex-associated CpGs from the models represented on the MSA array. This model generates a sex score by averaging the difference between male-associated hyper and hypo methylation over known sex-associated CpGs.

#### Linear modeling

Linear modeling for age and sex associated 5modC and 5hmC was performed using the *DML* function from the SeSAMe package^79^ version 1.22.0, covarying for tissue type (CpG ∼ Age + Sex + Tissue). *p*-Values were adjusted for multiple comparisons using the FDR method, and CpGs with FDR <0.05 for age and sex were considered for further analysis. Testis and placenta were excluded. For analysis of whole blood methylomes, cell type proportions from deconvolution analysis were regressed on the computed epigenetic age and sex (Cell proportion ∼ Age + Sex) using the linear modeling with lm() function in R.

#### Set enrichment analyses

All set enrichment analyses were performed using the *testEnrichmentSEA* function from the *knowYourCG* package R package (version 1.0.0). For testing epigenetic clock probes against 5hmC age probes, epigenetic clock probes were downloaded from the *dnaMethyAge* R package (https://github.com/yiluyucheng/dnaMethyAge) and tested against the ranked list of age-associated 5hmC probes, sorted according to *p* value from the 5hmC ∼ Age + Sex + Tissue EWAS. The top 10 most enriched clocks were plotted. For variable blood methylome analysis, autosomal probes were ranked according to the standard deviation across the 64 whole blood samples. EWAS trait CpGs^33^^,^^34^ were tested as queries against the variable probe list.

#### 5hmC/5mC/5modC age clocks

To compare the capacity of 5mC, 5hmC, and 5modC to predict age, leave-one-out cross-validation (LOOCV) approach was taken across all bulk tissue samples with matched 5modC/5hmC data except placental tissues. For each iteration of the LOOCV, one sample was withheld for testing, while the remaining samples were used for feature selection and model training. An EWAS was performed for each training set (Beta ∼ Age + Sex + Tissue Type), and the top 50 CpGs with the lowest P-values for Age were used for model training. Elastic net regression models were trained to predict age from the beta values using the *cv.glmnet* function (alpha = 0.5, nfolds = 10) from the *glmnet* package (Version 4.1–8). Mean absolute error (MAE) and the Pearson correlation were computed for all held-out test samples. This procedure was repeated for each cytosine modification type. A final model was re-trained on all samples.

#### Analysis of EWAS hit chromatin state contexts

Each set of EWAS trait probes in the curated studies was tested for enrichment in 100 full-stack ChromHMM chromatin states^53^ using Fisher’s exact test. The total pool of curated EWAS hits was used as a background set. The number of traits-chromatin state associations with FDR <0.05 was computed for each chromatin state and plotted. 6 major trait groups comprising 81 studies were selected, and the enrichment across chromatin states was plotted in heat maps.

#### Chromatin context analysis of EWAS methylations

The standard deviation of all probes was computed using the tissue methylomes generated on MSA and sorted to create a ranked probe list. Selected full-stack ChromHMM states were intersected with the list of total EWAS hits and tested as queries against the ranked probe list using a modified gene set enrichment approach^97^ using the *knowYourCG* R package (version 1.0.0).

#### Tissue context analysis of EWAS methylations

For each set of EWAS trait probes in the curated studies, we computed the standard deviation of the probes using the beta values from the tissue methylomes we generated using MSA. Trait sets were sorted according to the average standard deviations, and a subset of the most variable traits was selected for further analysis. In these trait groups, the rank for each sample was computed according to beta value. The mean rank of each tissue type group was computed for every CpG in the trait, and the distributions of ranks for each tissue type were plotted. A Kruskal-Wallis test was performed to test for differences in mean beta value rank distributions across tissue types. Dunn’s test was used for post-hoc testing. Summary statistics are available in Table S13.

#### GWAS co-localization with tissue-specific methylations

GWAS summary statistics were downloaded from the NHGRI-EBI GWAS catalog^102^ (version 1.0.2.1). The top 3000 unique disease/trait categories with the most SNPs were grouped and tested as independent queries against each one-vs-rest tissue/cell-specific CpG set from the curated lists incorporated into the final MSA design. SNPs and CpG sites were expanded by 5kbps in upstream and downstream directions, and genomic interval overlaps were computed using the *IRanges* package (version 2.36.0). The total number of CpG intervals for all tissue signatures was used as a background set, and Fisher’s Exact test was performed for enrichment testing.

## Supplemental information

Document S1. Figures S1–S7

Table S1. Datasets used in MSA design, related to Figure 1

Table S2. Trait associations and methylation biology annotations of designed MSA probes, related to Figure 1

Table S3. Comparison of MSA and EPICv2 probe locations in ENCODE *cis*-regulatory elements and chromatin states, related to Figure 1

Table S4. Nearest-neighbor reference for non-retained EPIC probes, related to Figure 1

Table S5. Cell-type proportion estimates and t-SNE coordinates for samples profiled on MSA, related to Figure 2

Table S6. Tissue-specific 5modC CpG signatures, related to Figure 2

Table S7. Tissue-specific 5hmC CpG signatures, related to Figure 4

Table S8. Tissue-specific CpG-Gene correlations, related to Figure 4

Table S9. CpGs with consistent hypo-, intermediate, or hyper-5modC across all tissues, related to Figure 5

Table S10. Age-associated 5modC and 5hmC CpG sites, related to Figure 5

Table S11. Epigenetic aging clocks for 5hmC, 5mC, and 5modC, related to Figure 5

Table S12. Sex-associated 5modC and 5hmC CpG sites, related to Figure 5

Table S13. Kruskal-Wallis and post hoc test statistics from tissue-type beta value ranks over trait-associated probes, related to Figure 6

Document S2. Article plus supplemental information
