# Supplementary material for: Scalable screening of ternary-code DNA methylation dynamics associated with human traits
Source: Cell Genom. 2025 Jul 3;5(9):100929. doi: 10.1016/j.xgen.2025.100929 (PMC12534708; doi:10.1016/j.xgen.2025.100929)
Supplement: Document S1. Figures S1–S7 [file mmc1.pdf]

Cell Genomics, Volume *5*

Supplemental information

Scalable screening of ternary-code DNA methylation dynamics associated with human traits

David C. Goldberg, Cameron Cloud, Sol Moe Lee, Bret Barnes, Steven Gruber, Elliot Kim, Anita Pottekat, Maximillian S. Westphal, Luana McAuliffe, Elisa Majounie, Manesh Kalayil Manian, Qingdi Zhu, Christine Tran, Mark Hansen, Jelena Stojakovic, Jared B. Parker, Rahul M. Kohli, Rishi Porecha, Nicole Renke, and Wanding Zhou

Figure S1, related to Figure 1

A

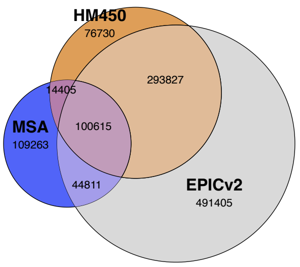

B

Total Probe Counts			
Type	EPICv2	MSA	MSA (Only)
CpG	933,252	274,661	120,522
CpH	2,914	2,781	2,482
Ctl	635	2,511	0
RS	65	4,364	3,970
NV	824	0	0

Unique Site Counts			
Type	EPICv2	MSA	MSA (Only)
CpG	926,732	262,467	117,432
CpH	2,914	2,776	2,482
Ctl	1	1	0
RS	62	3,850	3,788
NV	477	0	0

CpG = CpG probes; CpH = Cp(A/C/T) probes; Ctl = Control; RS = SNP probes; NV = Recurrent somatic mutation probes

C

EWAS Trait	Probe Count
Aging	106,954
Ancestry	33,168
Body Features	8,609
Cardiovascular	8,074
Environmental Exposure	46,199
Infectious Disease	16,136
Inflammation/Autoimmune	44,095
Lung/Respiratory	2,137
Metabolic	15,444
Neurodegenerative	4,894
Neurological/Neurodevelopmental	9,346
Psychiatric	7,444
Rare Genetic Disorder	14,118
Renal	1,356
Reproductive	7,435
Sex	25,196
Other	103,931

D

WGBS Design Group	Probe Count
5hmC	3,471
BLUEPRINT Signature	12,487
Brain Signature	16,307
CoRSIV	3,316
CpG-ATAC correlation	3,704
CpG-gene correlation	4,305
ENCODE CRE	28,754
GeneBody mCH Brain Signature	2,282
ImprintingDMR	123
IntermediateMeth	5,833
Loyfer Tissue Signature	15,060
SoloWCGW	5,143
XCILinked	4,792
Y-linked	1,837

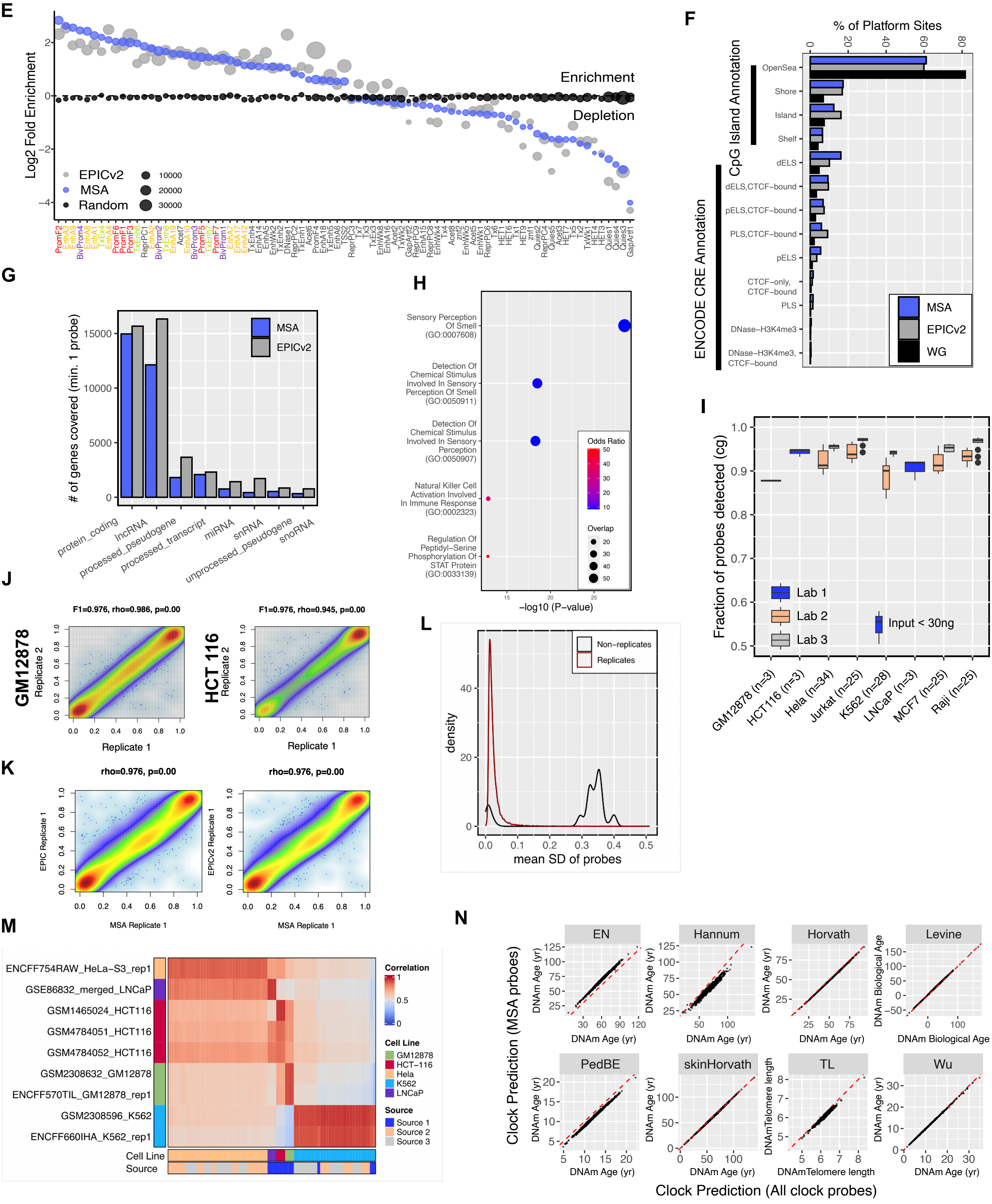

Figure S1: MSA probe selection summary and technical validation, related to Figure 1. (**A**) Venn diagram displaying the number of genomic sites covered on MSA and the overlap with EPICv2 and HM450 sites. (**B**) Table of total probe counts for different probe types on the EPICv2 platform, MSA platform, and the subset of novel probe designs on the MSA platform that are not found on previous Infinium arrays. Probe counts refer to total probe designs. Site counts refer to unique genomic loci targeted. There are more probes than total sites due to replicate probe designs that target the same loci (CpG = CpG probes, CpH = Cp(A|C|T) probes, Ctl=Control, RS=SNP probes, NV = Recurrent somatic mutation probes). (**C**) Table of probe counts for major EWAS trait group annotations. Probes are deduplicated within major trait groups. (**D**) Table showing deduplicated probe counts for whole genome methylation design groups targeted (**E**) Enrichment of MSA probes in full-stack ChromHMM states compared with EPICv2 probes and a random selection of genomic CpGs. (**F**) Percentage of sites in ENCODE cis-regulatory elements and CpG islands for MSA and EPICv2 compared to whole genome CpGs. (**G**) Number of genes covered with a minimum of one probe (within 1500bp of the TSS) for MSA and EPICv2. (**H**) Gene ontology results for genes not covered (minimum of one probe within 1500bp of TSS) on MSA. (**I**) Boxplots showing probe detection rates (y-axis) for different cell lines profiled on MSA (x-axis). The colors of the boxes indicate the laboratory of cell culture. (**J**) Scatter plots showing beta values for cell line technical replicates profiled on MSA. (**K**) Scatter plots showing beta values for cell line replicates profiled on EPIC (y-axis) and MSA (x-axis). (**L**) Density plots showing the distributions of within-sample mean standard deviations for replicate probe designs (red distribution) compared to within-sample mean standard deviations for non-replicate designs (black distribution). Replicate designs have lower variance compared to non-replicates. (**M**) Heatmap of beta value correlations between cell line samples profiled on MSA (columns) and publicly available WGBS data of related cell lines (rows). The first annotation bar represents the type of cell line, the second annotation bar for the columns indicates the laboratory of cell culture. (**N**) Correlation of epigenetic clock estimates using all clock probes (x-axis) and clock probes only on the MSA platform (y-axis) for 8 epigenetic clocks using TCGA normal tissue methylomes profiled on the HM450 array.

Figure S2, related to Figure 2

A

Tissue	Total	Sex (M/F)	5mC	5hmC
Adrenal	6	3/3	✓	✓
Bladder	6	3/3	✓	✓
CD4+ T cells	3	2/1	✓	✕
CD8+ T cells	3	2/1	✓	✕
Cerebellum	2	0/2	✓	✓
Colon	9	4/5	✓	✓
Esophagus	6	3/3	✓	✓
Eye	2	2/0	✓	✓
Heart	6	3/3	✓	✓
Kidney	5	2/3	✓	✓
Larynx	5	4/1	✓	✓
Liver	4	2/2	✓	✓
Lung	4	3/1	✓	✓
Lymph Node	3	1/2	✓	✓
Monocytes	3	2/1	✓	✕
Motor cortex	3	1/2	✓	✓
NK cells	3	1/2	✓	✕
Ovary	3	0/3	✓	✓
PBMC	3	2/1	✓	✕
Pancreas	6	4/2	✓	✓
Placenta	3	0/3	✓	✓
Prostate	4	4/0	✓	✓
Skin	6	3/3	✓	✓
Spinal Cord	1	0/1	✓	✓
Spleen	5	2/3	✓	✓
Stomach	6	3/3	✓	✓
Testis	6	6/0	✓	✓
Thymus	6	3/3	✓	✓
Thyroid	5	3/2	✓	✓
Total T cells	3	2/1	✓	✕
Trachea	5	3/2	✓	✓

B

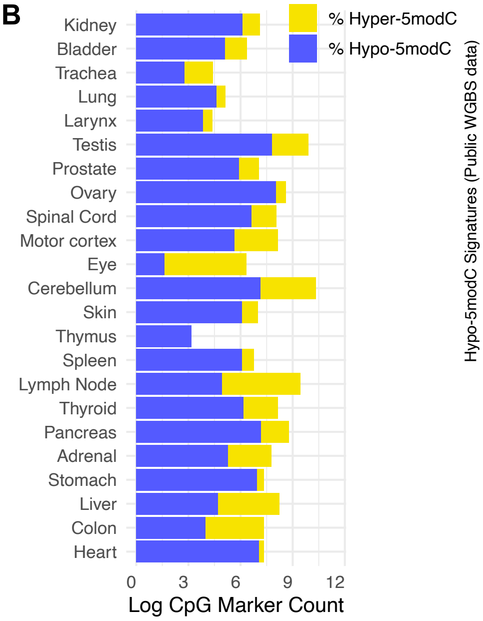

C

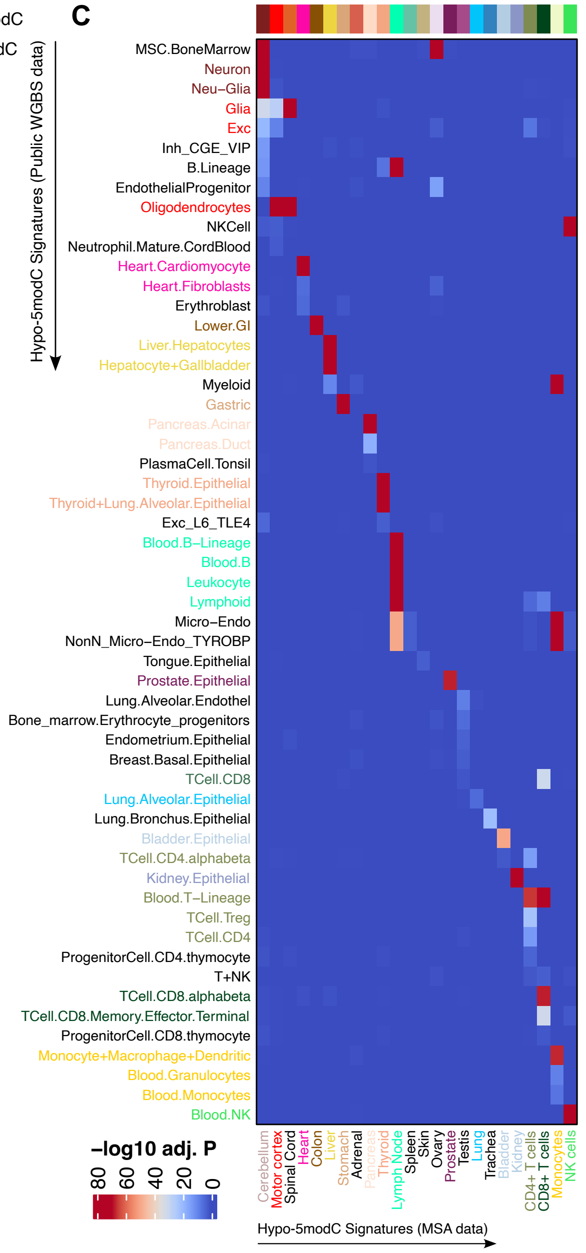

D

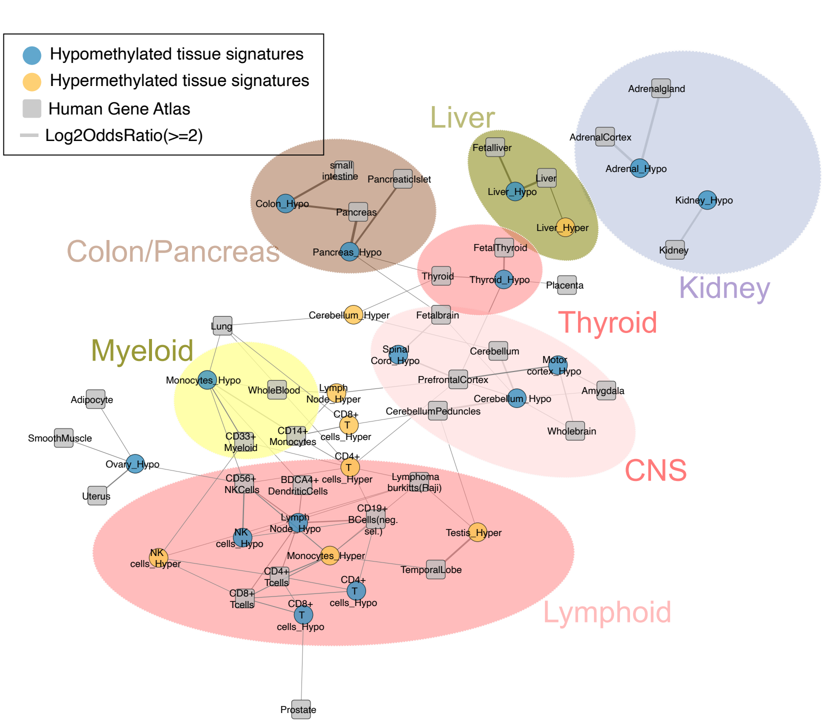

E

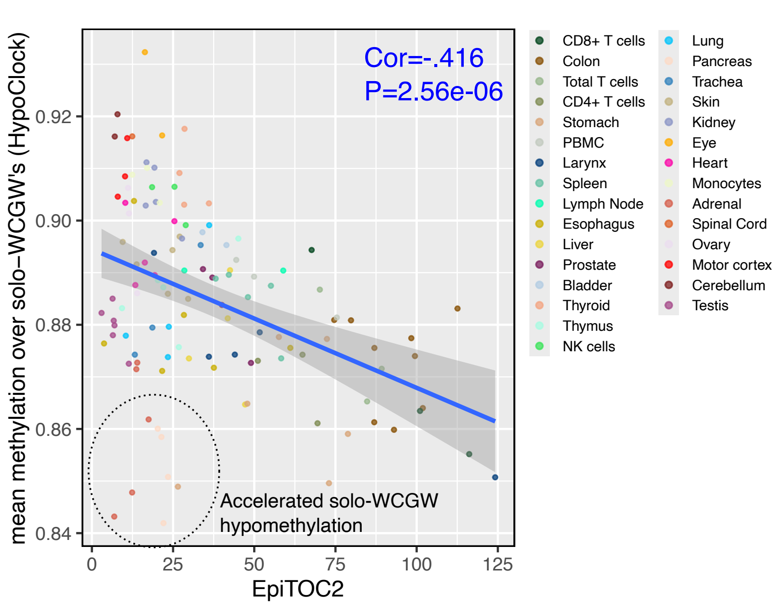

F

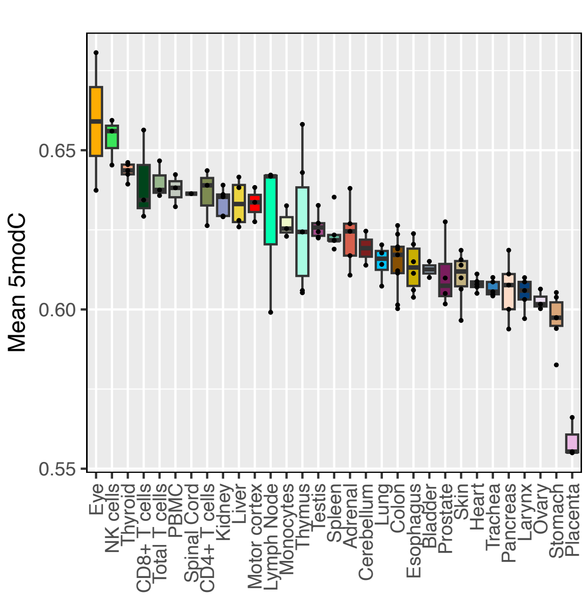

Figure S2: Tissue-specific methylation detected on MSA, related to Figure 2. (**A**) Table of human tissues profiled on MSA for 5modC and 5hmC in this study. (**B**) Log counts of tissue-specific 5modC markers identified for tissues profiled in the study. The heights of the bars indicate the log of the total signature counts. The blue portion is the percentage of the total that are hypo-5modC, the yellow portion is the percentage of the total that are hyper-5modC. (**C**) Heatmap showing enrichment of tissue-specific hypo-5modC CpGs identified in the current study using MSA (columns) in cell-specific hypo-5modC CpG curations from publicly available WGBS data (rows). Colors of labels on axes correspond to tissue type. (**D**) Network showing connections of gene sets linked to tissue-specific CpGs identified in this study (blue and orange nodes) with tissue-specific genes from the Human Gene Atlas (grey nodes). Edges represent the enrichment of gene sets. Yellow nodes indicate gene sets linked to hyper-5modC signatures while blue nodes are gene sets linked to hypo-5modC signatures. Colors outlining communities relate to tissue type. (**E**) Pearson correlation of cell division estimates computed with EpiTOC2 (x-axis) and solo-WCGW methylation (y-axis) for MSA profiled tissues. Circled tissue types (adrenal, pancreas) show accelerated solo-WCGW hypomethylation relative to EpiTOC2. (**F**) Mean CpG 5modC levels for tissues profiled with MSA in this study.

Figure S3, related to Figure 3

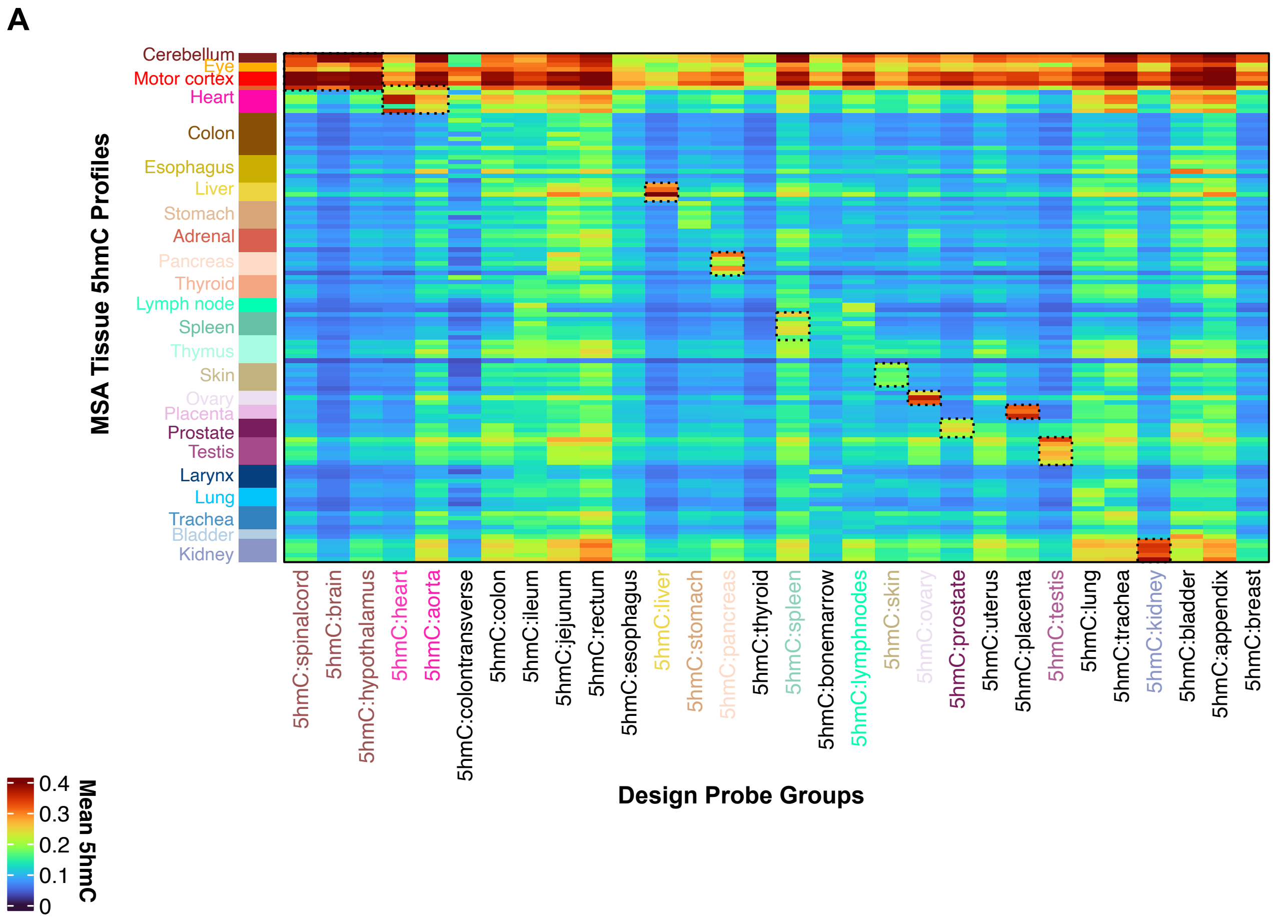

Figure S3: Analysis of global 5hmC across human tissues with MSA, related to Figure 3. (**A**) Heatmap showing mean 5hmC levels for each sample (rows) across the probe sets for the 5hmC design groups created during array development (columns). Colors of labels on axes correspond to tissue type.

Figure S4, related to Figure 4

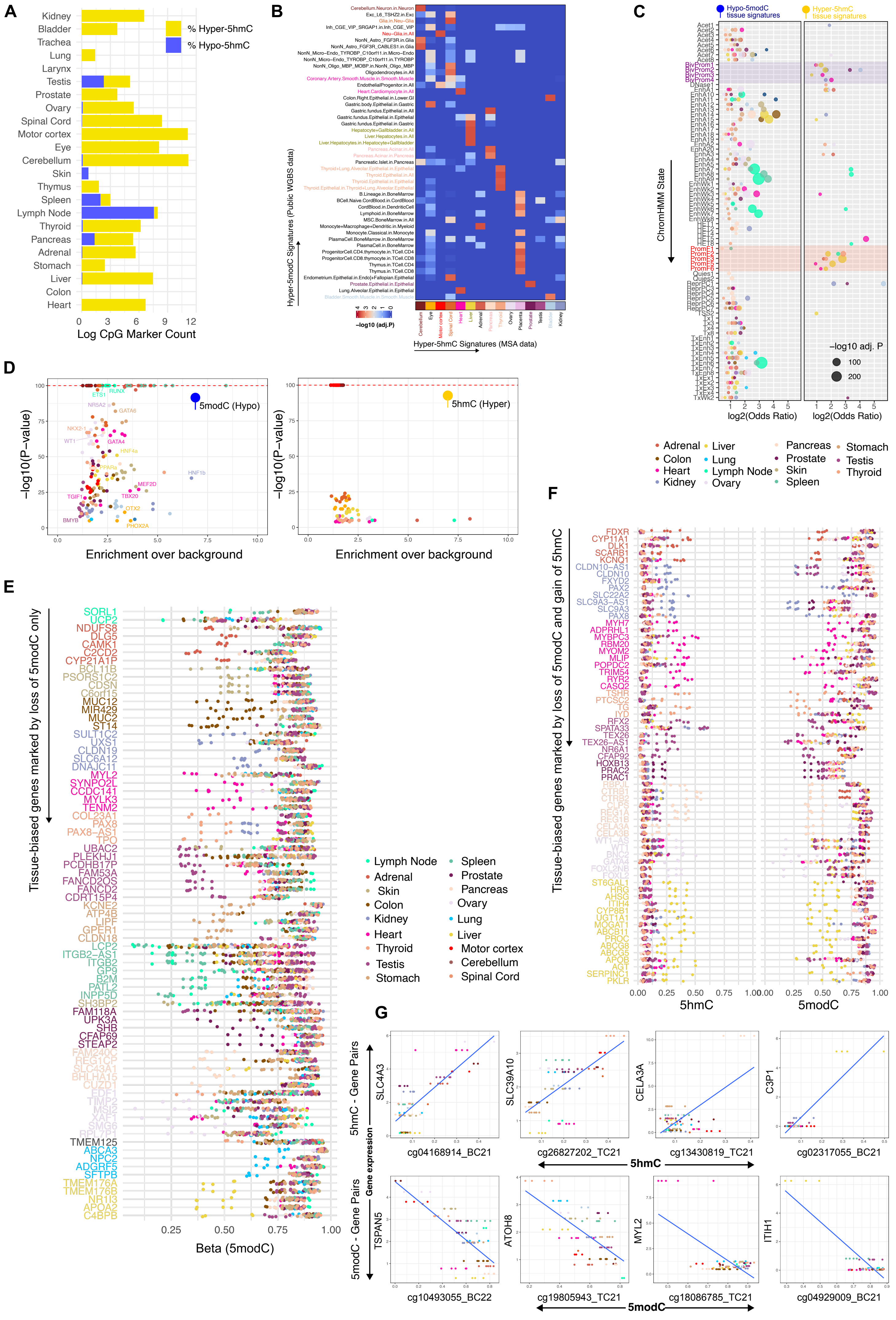

Figure S4: MSA reveals the role of 5hmC in defining human tissue identity, related to Figure 4. (**A**) Log counts of tissue-specific 5hmC markers identified for tissues profiled in the study. The heights of the bars indicate the log of the total signature counts. The blue portion is the percentage of the total that are hypo-5hmC, the yellow portion is the percentage of the total that are hyper-5hmC. (**B**) Heatmap showing enrichment of tissue-specific hyper-5hmC CpGs identified in the current study using MSA (columns) in cell-specific hyper-5modC CpG curations from publicly available WGBS data (rows). Colors of labels on axes correspond to tissue type. (**C**) Enrichment of tissue-specific hypo-5modC probes (left) and tissue-specific hyper-5hmC probes (right) identified in the study in full-stack ChromHMM states (**D**) Enrichment of tissue-specific hypo-5modC signatures (left) and tissue specific hyper-5hmC signatures (right) identified in the study in transcription factor binding motifs from HOMER analysis. Points are colored by tissue type. Colored labels are representative results where the tissue-specific CpG sets were enriched in a tissue-specific transcription factor binding motif from the same tissue. (**E**) Representative genes showing tissue-biased expression that are marked only by tissue-specific hypo-5modC. The x-axis is the averaged 5modC over the tissue-specific probes linked to each gene on the y-axis. The y-axis genes are colored according to the tissue type that has biased expression of the gene. (**F**) Representative genes showing tissue-biased expression that are marked by tissue-specific hypo-5modC and tissue-specific hyper-5hmC at the same gene. The x-axis is the averaged 5hmC over tissue-specific 5hmC probes (left) and averaged 5modC over the tissue-specific 5modC probes (right) for each gene on the y-axis. The y-axis genes are colored according to the tissue type that has biased expression of the gene. (**G**) Correlations of beta values (x-axis) with gene expression (y-axis) from publicly available data for matching tissue types. The top plots show 5hmC – gene correlations while the bottom are 5modC – gene correlations.

Figure S5, related to Figure 5

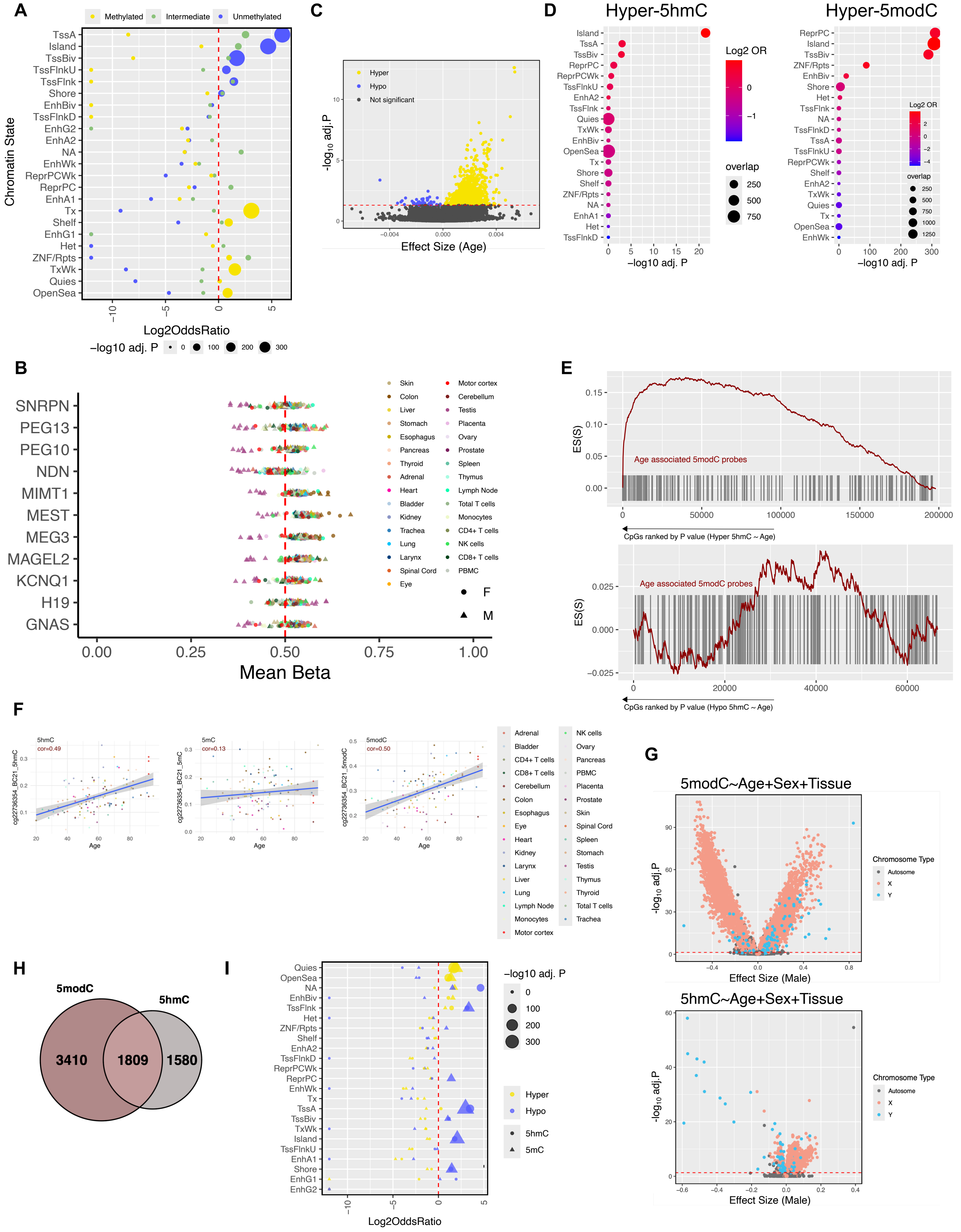

Figure S5: 5modC and 5hmC methylation biology in imprinting, aging, and sex specificities, related to Figure 5. (**A**) Dotplot showing enrichment of constitutive hypo, hyper, or intermediately modified CpG classes across consensus ChromHMM states. (**B**) Mean 5modC over intermediately methylated probes linked to known imprinting genes. (**C**) Volcano plot showing CpGs with 5modC levels associated with aging from the 5modC aging EWAS. Yellow dots are CpGs that gain 5modC with aging, while blue dots are CpGs that lose 5modC with aging. (**D**) Dot plot showing enrichment of age-associated hyper-5hmC CpGs (left) and age-associated hyper-5modC (right) in consensus ChromHMM states. (**E**) Set enrichment analysis showing enrichment of age-associated 5modC probes in the ranked list of age-associated hyper-5hmC probes (top) and age-associated hypo-5hmC probes (bottom). Hyper-5hmC and hyper-5modC aging probes are mutually enriched while hypo-5hmC are not. (**F**) Scatter plots showing the relationship between beta value and age at an age- associated probe. Left is 5hmC and age, middle is 5mC and age, right is 5modC and age. 5hmC and 5modC associate with aging at this probe, but not 5mC. (**G**) Volcano plots for the Sex EWAS' performed in the study for 5modC (top panel) and 5hmC (bottom panel). Orange points are X-chromosome probes that significantly associate with sex, blue points are Y-chromosome probes and gray points are autosomal probes. The dashed red line indicates statistically significant probes. (**H**) Venn diagram showing overlap of sex-associated 5hmC and 5modC probes (**I**) Dot plot showing enrichment of sex-associated 5modC and 5hmC across consensus ChromHMM states. The shape of the points indicates whether the query are 5hmC probes (circle) or 5modC probes (triangle) and the color represents whether the probes are hyper (yellow) or hypo-modified (blue) with sex.

Figure S6, related to Figure 6

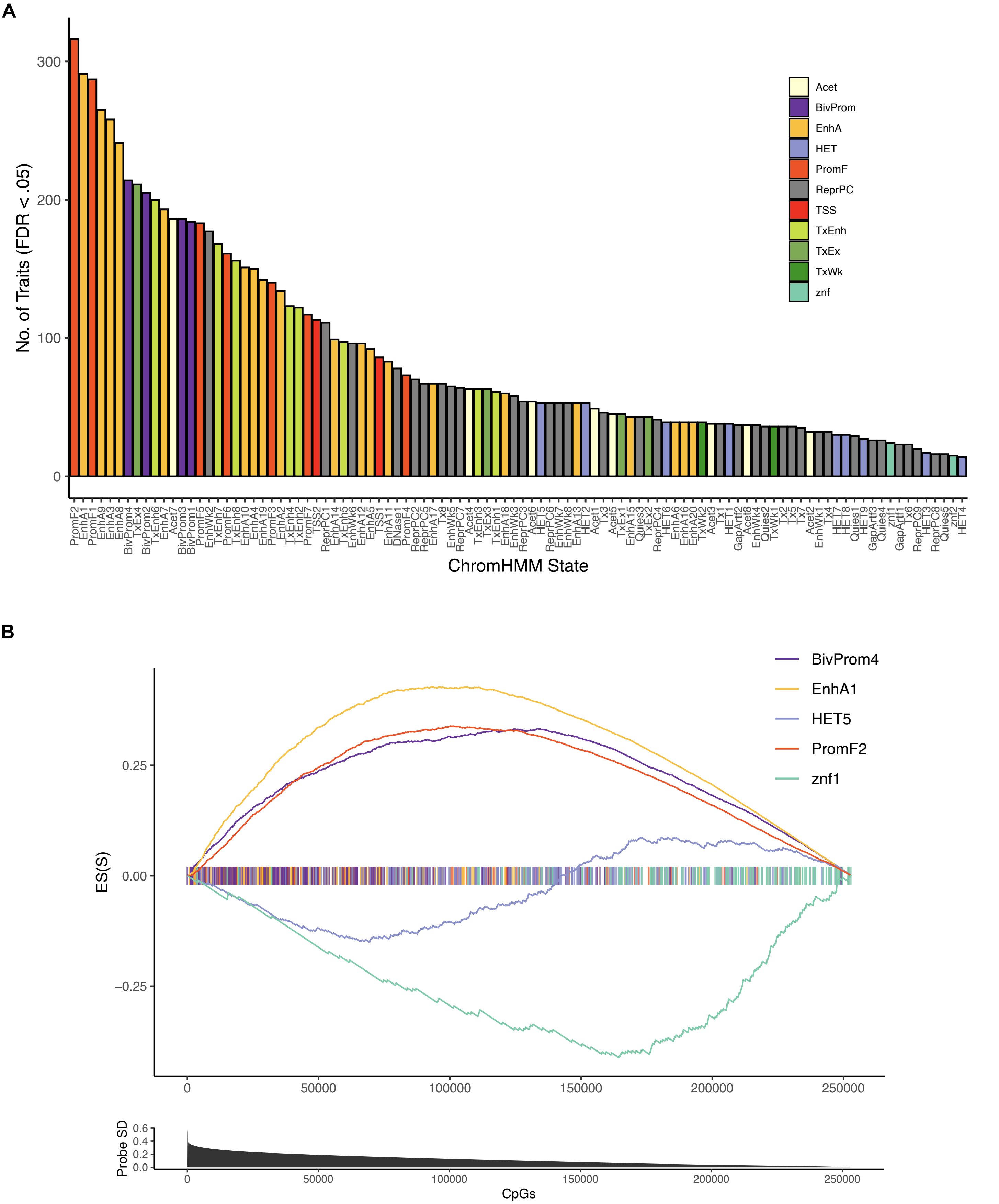

Figure S6: Tissue and chromatin context of human trait associations, related to Figure 6. (**A**) The number of EWAS traits significantly enriched for each full-stack ChromHMM chromatin state (x-axis). Color of bars represents the chromatin state. (**B**) Set enrichment plot showing where EWAS hits from the selected chromatin states are enriched in the ranked list of most variable probes in MSA-profiled human tissues.

Figure S7, related to Figure 7

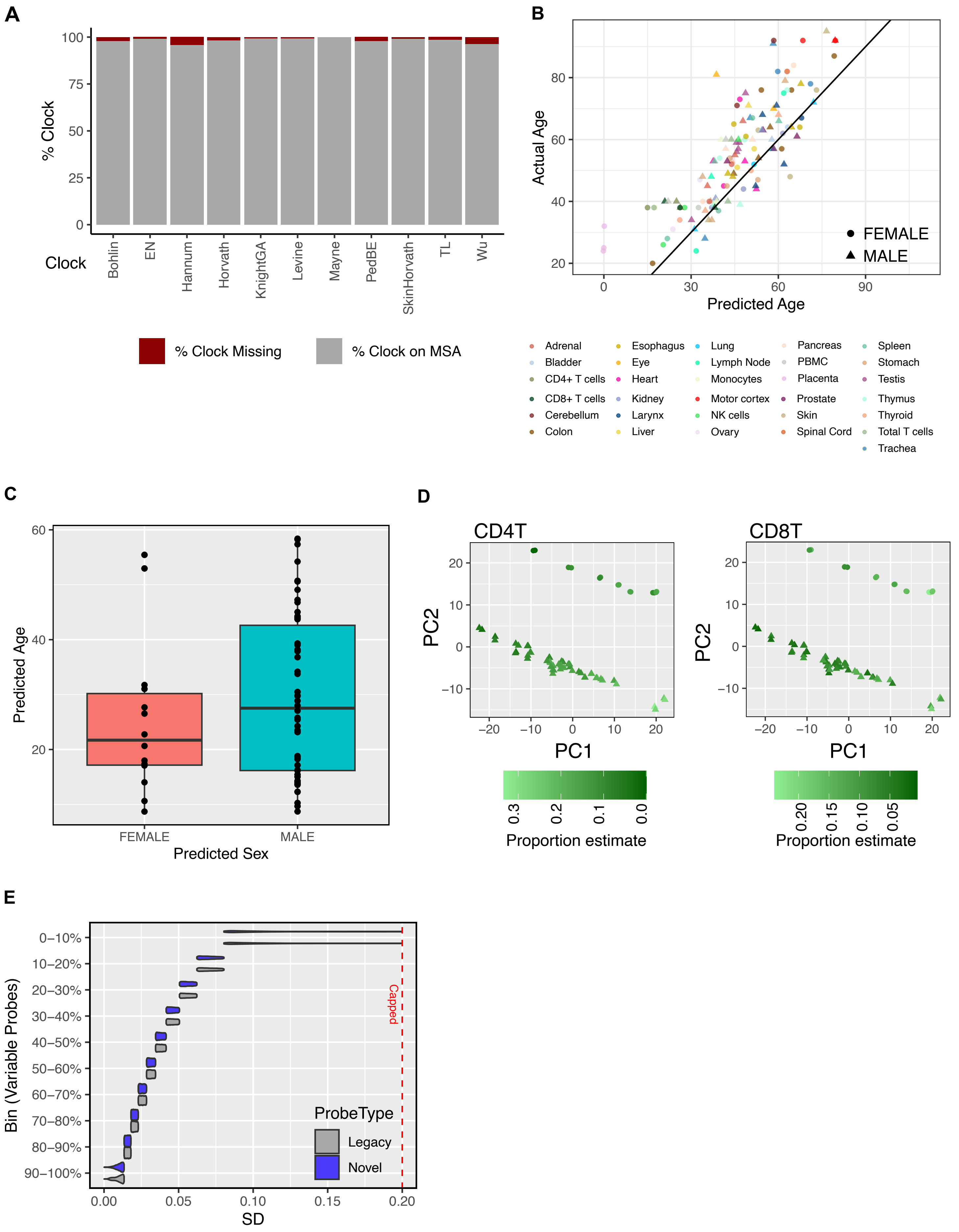

Figure S7: Immune cell composition and interindividual whole blood methylation variation, related to Figure 7. (**A**) Stacked bar plots showing the percent of probes covered on MSA (grey) and the percent missing (red) for 11 epigenetic clocks (**B**) Horvath 5modC-based age estimates (x-axis) compared to reported age for the tissues profiled on MSA. Age estimates are highly correlated with reported age (Pearson correlation=0.82) (**C**) Boxplots showing the distribution of sex and age predictions from MSA-profiled whole blood samples. (**D**) PCA plot of whole blood methylomes with CD4T (left) or CD8T (right) cell type proportions overlaid. (**E**) Violin plots showing distributions of novel probe designs and reintroduced legacy probes over each decile of variably methylated probes. Novel probe designs detect similar variations.
